# N6-methyladenosine-associated prognostic pseudogenes contribute to predicting immunotherapy benefits and therapeutic agents in head and neck squamous cell carcinoma

**DOI:** 10.7150/thno.76689

**Published:** 2022-10-17

**Authors:** Liqiang Tan, Yali Qin, Ruiling Xie, Tianliang Xia, Xiaotong Duan, Lan Peng, Rui You, Youping Liu, Xiong Zou, Mengxia Zhang, Mei Lin, Mingyuan Chen

**Affiliations:** 1 Department of Nasopharyngeal Carcinoma, Sun Yat-sen University Cancer Center, No.651 Dongfeng East Road, Guangzhou 510060, China.; 2 Sun Yat-sen University Cancer Center, State Key Laboratory of Oncology in South China, Collaborative Innovation Center for Cancer Medicine, Guangzhou 510060, China.; 3 Guangdong Key Laboratory of Nasopharyngeal Carcinoma Diagnosis and Therapy, Guangzhou 510060, China.; 4 Department of Ophthalmology, The Second Clinical College of Guangzhou University of Chinese Medicine, Guangdong Provincial Hospital of Chinese Medicine, Guangzhou 510120, China.

**Keywords:** N6-methyladenosine, Pseudogene, Immunotherapy benefits, Therapeutic agents, Head and neck squamous cell carcinoma

## Abstract

**Rationale:** N6-methyladenosine (m^6^A) is involved in critical cancerous processes. Pseudogenes play various roles in carcinogenesis and progression. However, the functional roles of m^6^A-associated pseudogenes in head and neck squamous cell carcinoma (HNSCC) are largely unknown.

**Methods:** We systematically analyzed the mRNA profile of 24 m^6^A regulators and 13931 pseudogenes from The Cancer Genome Atlas HNSCC dataset and ultimately identified 10 m^6^A-associated prognostic pseudogenes, which were validated in the Gene Expression Omnibus and our hospital datasets. Based on the risk score of m^6^A-associated pseudogenes, comprehensive analytical frameworks and experimental validation were implemented among pseudogene-defined low-/high-risk subtypes.

**Results:** Here, we found expression pattern of m^6^A-associated pseudogenes was significantly associated with infiltrating immune cell compositions, and the expression of antitumor immune response markers, including T cell exhaustion, antigen presentation, interferon, and kinase genes. The m^6^A-associated pseudogenes, which had dramatic m^6^A peaks and higher m^6^A levels, could regulate the expression of targeted immune-involved genes through miRNAs. We experimentally validate the oncogene PDIA3P1, and tumor-suppressor RRN3P3, which promote the RNA and protein expression of their targeted immune-involved genes AKT1 and EZH2 via miR-34a-5p and miR-26b-5p, respectively. Moreover, HNSCC patients in the high-risk subtype could benefit more from immune checkpoint inhibitors therapy. Furthermore, doxorubicin and topotecan were considered to hold the most promising therapeutic potential robustly in silico evidence and *in vitro* experiments for HNSCC patients in the high-risk subtype.

**Conclusions:** Our discovery revealed that the 10 m^6^A-associated prognostic pseudogenes significantly contribute to predicting immunotherapy benefits and therapeutic agents, which might bring some potential implications for both immunotherapy and chemotherapy in HNSCC.

## Introduction

Head and neck squamous cell carcinoma (HNSCC) accounts for over 900,000 cases and approximately 400,000 deaths per year worldwide 1, which is characterized by aetiological, phenotypic, and clinical heterogeneity. Smoking causes the rise of HNSCC in developing countries, and human papillomavirus (HPV) is emerging as an important factor in the rise of oropharyngeal tumors of non-smokers in developed countries 2. Although advances in surgery, radiation, and chemotherapy, around half of all patients, will die of the disease. Despite risk stratification for HNSCC by age, gender, anatomic site, TNM stage, HPV status, laterality, and histological characteristics, numerous molecular biomarkers that have been investigated have limited clinical utility. Therefore, seeking new promising prognostic biomarkers at the intrinsic molecular level is necessary, which contributes to identifying high-risk subtypes and making precise therapeutic strategies.

Except for surgery, radiotherapy, chemotherapy, and targeted therapy based on molecular subtypes and the TNM stage of HNSCC, immunotherapy is an emerging treatment modality due to the promising therapeutic effect of selective immune checkpoint inhibitors (ICI), including monoclonal antibodies against programmed death 1 (PD-1), programmed death-ligand 1 (PD-L1) and cytotoxic T lymphocyte-associated protein-4 axes (CTLA-4) 3-5. These expression levels of PD-1, PD-L1, and CTLA-4 in tumor tissue are currently used as predictive markers for immune response. And these predictive results were still not satisfactory, indicating that immune modulation in tumor tissue is a complex process and needs many more functional predictors 6. Thus, it is necessary to identify new robust predictive biomarkers for the immune response process when executing clinical trials of immunotherapy.

Pseudogenes played a pivotal role in many human diseases, including tumorigenesis and tumor progression 7, 8. As non-functional homologs of protein-coding genes, Pseudogenes are commonly caused by the accumulation of multiple nonsense mutations within genes. Based on the unique biogenesis mechanisms, pseudogenes are divided into three categories: unitary pseudogenes, unprocessed pseudogenes, and processed pseudogenes 9. Although pseudogenes were once considered “genetic fossils” due to their lack of protein-coding ability or cellular gene expression, increasing evidence indicates that some pseudogenes have been identified to participate in multiple biological functions and regulate their parental transcripts by acting as competitive endogenous RNAs (ceRNA) 10. What's more, dysregulation of pseudogenes is associated with diseases, which was immensely attributed to the discovery that the PTENP1 11 and BRAFP1 12 could upregulate their corresponding parental gene PTEN and BRAF via the ceRNA mechanism.

N6-methyladenosine (m^6^A) RNA modification is pervasively a reversible internal RNA modification in most kinds of RNAs coving mRNAs and long noncoding RNAs (lncRNAs), which has been confirmed to participate in regulating RNA transcription, processing, and so on 13. It is installed by m^6^A methyltransferases (termed as “writers”) complicated with METTL3 as the catalytic subunit and reversed by demethylases (termed as “erasers”) FTO and ALKBH5 14, 15. In addition, m^6^A can be specifically regulated through a series of RNA binding proteins (termed as “readers”) and co-transcriptionally through a variety of transcription factors 14, 15. As m^6^A readers, YTH domain-containing proteins can specifically read the m^6^A and regulate diverse post-transcriptional processes of host mRNAs 14, 15. Accumulating evidence demonstrated that critical roles of m^6^A have been identified in the development of multiple cancers 16.

According to the genome-wide profiling of m^6^A, pseudogenes are also modified by m^6^A in non-tumor cell lines such as GM12878 and H1 17. Our previous research found that pseudogenes and m^6^A were significantly correlated with host antitumor immune response and might serve as potential biomarkers for immunotherapy in breast cancer 18, 19. Furthermore, we investigated the function of the m^6^A sites on pseudogenes in non-tumor cell lines, which indicated a new evolutionary role of m^6^A in cleaning up the unnecessary processed pseudogenes to mitigate their interference of expression of cognate protein-coding genes 20. However, little is known about what the function of m^6^A-associated pseudogenes is and whether they can predict immunotherapy benefits and therapeutic agents in HNSCC.

In this study, as shown in the workflow (Figure S1), we systematically analyzed the RNA sequencing data of HNSCC patients and eventually identified and validated 10 m^6^A-associated prognostic pseudogenes, which were independently prognostic indicators in HNSCC patients. We further found a close association between the m^6^A-associated prognostic pseudogenes and the antitumor immune response from different aspects. Our convergent evidence showed that HNSCC patients could benefit more from immune checkpoint inhibitor therapy and the most promising potential therapeutic agents (doxorubicin and topotecan) for HNSCC patients. Our study indicated the 10 m^6^A-related prognostic pseudogenes could predict immunotherapy benefits and potential therapeutic agents in HNSCC.

## Results

### Identification and validation of 10 m^6^A-associated prognostic pseudogenes in TCGA and GEO datasets

**Figure 1:**
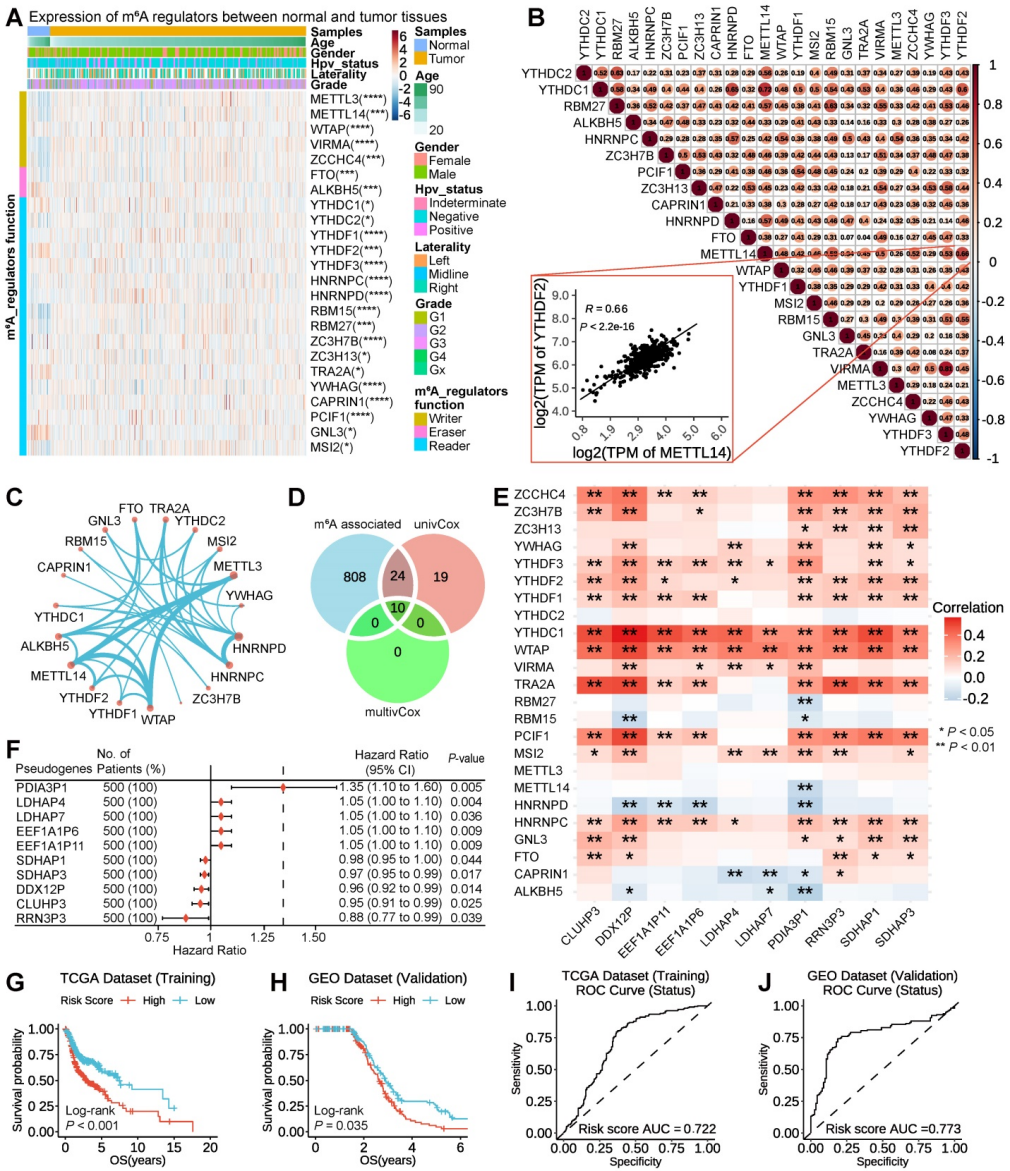
** Identification and validation of 10 m^6^A-associated prognostic pseudogenes in TCGA and GEO datasets. (A)** Heatmap showing the expression of 24 m^6^A regulators across different clinicopathological features between normal tissues and tumor tissues. The expression value between normal and tumor tissues were compared through the Wilcoxon test. ns denotes no significance, * denotes *P* < 0.05, ** denotes *P* < 0.01, *** denotes *P* < 0.001 and **** denotes *P* < 0.0001. **(B)** Triangle heatmap of Pearson correlation among the expression of m^6^A regulators. The scatter plot shows the correlation of the expressions between *METTL14* and *YTHDF2*. **(C)** Circular arc diagram of the protein-protein interactions among m^6^A regulators. **(D)** Venn diagram revealing the overlapping m^6^A-associated prognostic pseudogenes screened by three methods. **(E)** Heatmap showing Pearson correlation of the expression between 24 m^6^A regulators with 10 m^6^A-associated prognostic pseudogenes in TCGA dataset. **(F)** Forest plot of the hazard ratios (HR), 95% confidence intervals (CI) calculated by univariate Cox proportional hazard regression of 10 m^6^A-associated prognostic pseudogenes using TCGA dataset. **(G)** Kaplan-Meier survival curve reveals that the HNSCC patients from TCGA dataset in the high-risk group displayed significantly shorter overall survival than those in the low-risk group (*P* < 0.001). **(H)** Kaplan-Meier survival curve reveals that the HNSCC patients from the GEO dataset in the high-risk group displayed significantly shorter overall survival than those in the low-risk group (*P* = 0.035). **(I)** The ROC curve shows AUC for the risk score model in the TCGA dataset. **(J)** The ROC curve shows AUC for the risk score model in the GEO dataset.

To study the functions of m^6^A-related pseudogenes in HNSSC, we first ask whether m^6^A regulators are expressed differently between HNSSC tissues with normal tissues. We collected 24 m^6^A regulators from studies that have been publicly reported (List S1) and explored their expression between 500 tumor tissues and 44 normal tissues in 500 HNSSC patients with different clinicopathological features. Our research results showed that the expressions of m^6^A regulators differed enormously across subtypes (Figure 1A). For example, compared to normal tissues, the expressions of *METTL3*, *METTL14*, *WTAP*, *VIRMA*, *ZCCHC4*, *FTO*, *ALKBH5*, *YTHDC1*, *YTHDF1*, *YTHDF2*, *YTHDF3*, *HNRNPC*, *HNRNPD*, *RBM15*, *RBM27*, *ZC3H7B*, *YWHAG*, *CAPRIN1* and *PCIF1* increased significantly in tumor tissues, while the expressions of *YTHDC2*, *ZC3H7B*, *TRA2A*, *GNL3*, and *MSI2* decreased (Figure S2). Besides, the expression pattern of m^6^A regulators was inconsistent among different clinicopathological features, which indicated the crucial but complicated roles of m^6^A regulators in tumorigenesis and the development of HNSCC (Figure 1A). On account, genes do not perform a function in isolation, and increasing evidence has manifested that collaboration among writers, erasers, and readers exists in the context of cancer. Thus the co-expression among 24 m^6^A regulators by Pearson correlation analysis was investigated. We found not only that m^6^A regulators within the same functional class showed significant co-expression and highly correlated expression patterns, but that a close correlation also existed among writers, erasers, and readers (Figure 1B). For instance, the reader YTHDF2 was significantly correlated with writers, such as METTL14 (Figure 1B, *R* = 0.66 and *P*-value < 2.2e-16). Through the String database, we found that these 5 writers, 2 erasers, and 17 readers interacted with each other frequently in protein-protein interaction networks (Figure 1C). The high degree of co-expression and strong interaction among 24 m^6^A regulators suggested that m^6^A RNA modification played an important and complex role in HNSCC.

Then, a total list of 13931 pseudogenes was compiled from GENCODE, Vega, and psiCube databases (List S2), of which 6218 pseudogenes were obtainable in TCGA HNSCC datasets and thus used in the subsequent analyses. Firstly, we took advantage of the Pearson correlation analysis of expression between candidate pseudogene with 24 m^6^A regulators to filter a list of 2682 one-to-one pairs of pseudogenes and m^6^A regulators at | *R* | ≥ 0.3 and *P* < 0.05 (Table S1), including 842 pseudogenes with different numbers of m^6^A regulators. Secondly, through the univariate Cox proportional hazard regression, 53 pseudogenes were screened at *P* < 0.05 (Table S2). Thirdly, based on the second step analysis above, the multivariate Cox proportional hazard regression was implemented. Using the above three methods, overlapping 10 m^6^A-associated prognostic pseudogenes (*PDIA3P1*, *LDHAP4*, *LDHAP7*, *EEF1A1P6*, *EEF1A1P11*, *SDHAP1*, *SDHAP3*, *DDX12P*, *CLUHP3*, *RRN3P3*) were ultimately identified (Figure 1D).

To further interpret the molecular mechanisms by which m^6^A regulators and pseudogenes are involved in cancer, we examined the Pearson correlation between the expression of individual m^6^A regulators and the 10 m^6^A-associated prognostic pseudogenes. We found that there were significantly higher correlations between *YTHDC1* and* WTAP* with 10 prognostic pseudogenes, which implies that *YTHDC1* and *WTAP* play important roles in the function of m^6^A modification on these 10 pseudogenes (Figure 1E). Forest plot of 10 m^6^A-associated prognostic pseudogenes showed that the hazard ratio of *PDIA3P1*, *LDHAP4*, *LDHAP7*, *EEF1A1P6*, *EEF1A1P11* was greater than 1, which indicated that these 5 pseudogenes may be factors with poor prognosis, while the hazard ratio of *SDHAP1*, *SDHAP3*, *DDX12P*, *CLUHP3*, *RRN3P3* was less than 1, hinting these 5 pseudogenes may be factors with good prognosis (Figure 1F). What's more, these 10 m^6^A-associated prognostic pseudogenes were further verified in the GEO GSE65858 dataset (Figure S3A). To raise the predictive effect of pseudogenes in the clinical outcomes of HNSCC, we applied the least absolute shrinkage and selection operator (LASSO) Cox regression algorithm to the 10 m^6^A-associated prognostic pseudogenes and established a risk signature based on the minimum criteria using TCGA HNSCC data as the training set (Figure S3B-C) and GEO GSE65858 data as the validation set. Based on the coefficients of the 10 pseudogenes (Table S3), the risk score was calculated on account of the survival risk score model formula. Then, according to the median risk score, the HNSCC patients were dichotomized into low or high-risk groups. We found that patients in the high-risk subtype displayed remarkably shorter overall survival than those in the low-risk subtype (TCGA dataset, log-rank test, *P* < 0.001, Figure 1G; GEO dataset, log-rank test, *P* = 0.035, Figure 1H). The patients in high-risk group were significantly associated with female (*P* = 0.008), higher Pathologic T stage (*P* = 0.003) and higher Pathologic M stage (*P* = 0.040) (Table S4). In addition, to better understand whether m^6^A-associated pseudogenes be able to effectively predict the prognosis of HNSCC patients, the receiver operating characteristic curve (ROC) curve analysis was performed. Results showed that the risk score based on m^6^A-associated pseudogenes was a good predictor of survival rates with an area under the curve (AUC) value of 0.722 in the training set (Figure 1I) and 0.773 in the validation set (Figure 1J).

### Screened 10 m^6^A-associated pseudogenes are an independent prognostic factor in HNSCC patients

**Figure 2:**
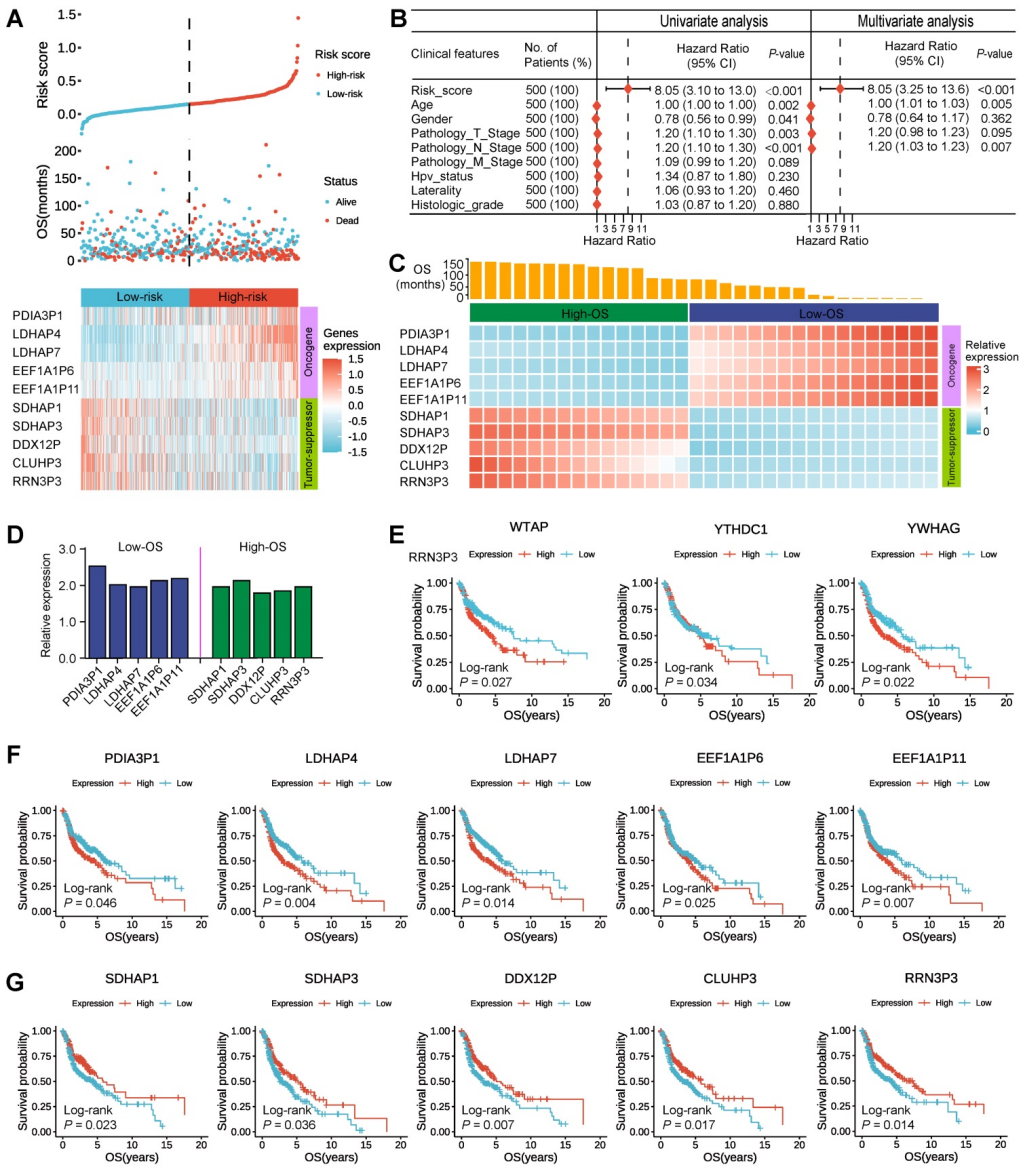
** Screened 10 m^6^A-associated pseudogenes are an independent prognostic factor in HNSCC patients. (A)** The composite plot of the distribution of risk score, vital status, and the expression pattern of 10 m^6^A-associated prognostic pseudogenes in 500 HNSCC patients. The risk scores are arranged in ascending order from left to right. **(B)** Forest plot of univariate and multivariate Cox regression analyses of the association between clinicopathological factors (including the risk score) and overall survival (OS) of HNSCC patients. ns denotes no significance, * denotes *P* < 0.05, ** denotes *P* < 0.01, *** denotes *P* < 0.001 and **** denotes *P* < 0.0001. **(C)** The heatmap of the relative RNA expression (by qPCR) of 10 m^6^A-associated pseudogenes and OS of the corresponding patient in 32 HNSCC tissues from the Tumor Resource Bank of Sun Yat-sen University Cancer Center. **(D)** Barplot of the mean relative RNA expression of oncogene pseudogenes in 16 low-OS subtype tissues and tumor-suppressor pseudogenes in 16 high-OS subtype tissues. **(E)** Kaplan-Meier curves of association between the expression levels of m^6^A regulators and overall survival in patients with HNSCC from TCGA dataset. **(F)** Kaplan-Meier curves of association between the expression levels of oncogene pseudogenes and overall survival in patients with HNSCC from TCGA dataset. **(G)** Kaplan-Meier curves of association between the expression levels of tumor-suppressor pseudogenes and overall survival in patients with HNSCC from TCGA dataset.

To intuitively understand the prognostic effect of m^6^A-associated pseudogenes, the distribution of the risk scores based on pseudogenes, overall survival of HNSCC, and corresponding pseudogene expression profiles in the TCGA dataset were displayed (Figure 2A). The composite plot indicated that *SDHAP1*, *SDHAP3*, *DDX12P*, *CLUHP3*, and *RRN3P3* demonstrated high expressions in the low-risk subtype, which were categorized as tumor-suppressor pseudogenes in our study. However, the residual pseudogenes (*PDIA3P1*, *LDHAP4*, *LDHAP7*, *EEF1A1P6*, and *EEF1A1P11*) displayed high expressions in the high-risk subtype and therefore were classified as oncogene pseudogenes in our study (Figure 2A). Besides, our study also revealed that the risk score and prognostic pseudogenes were significantly related to different clinicopathological features of HNSCC patients. Compared to HPV negative, patients with HPV positive had significantly higher expressions of oncogene pseudogenes (*SDHAP1*, *SDHAP3*, *DDX12P*, *CLUHP3*, and *RRN3P3*), but there was no prominent difference in the expressions of tumor-suppressor pseudogenes (*PDIA3P1*, *LDHAP4*, *LDHAP7*, *EEF1A1P6*, and *EEF1A1P11*) (Figure S4A). In addition, patients with higher grades (such as G4) had significantly higher expressions of *PDIA3P1*, *EEF1A1P6*, *EEF1A1P11*, *SDHAP1*, *SDHAP3*, *DDX12P*, *CLUHP3*, and *RRN3P3*, but lower expressions of *LDHAP4* and *LDHAP7* than those with lower grades (Figure S4B). On the contrary, there were no striking differences in the expressions of the 10 m^6^A-associated prognostic pseudogenes among patients with different TNM (Figure S5A-C).

On account of a series of factors, such as HPV status and different clinical features, which can affect the prognosis of HNSCC patients, we couldn't help asking whether m^6^A-associated prognostic pseudogenes are independent prognostic factors. To address the doubt, univariate and multivariate Cox regression analyses are simultaneously executed. Then our findings demonstrated that risk score, age, Gender, pathology T stage, and pathology N stage were all correlated with the overall survival by univariate Cox regression analysis. What's more, when including these factors in the multivariate Cox regression, our analysis revealed that risk score (*P* < 0.001), age (*P* = 0.005), and pathology N stage (*P* = 0.007) remained closely associated with the prognosis (Figure 2B), which proved that the risk score derived from these 10 m^6^A-associated pseudogenes was able to independently predict the clinical outcome in HNSCC patients.

To further demonstrate the prospect of m^6^A-associated pseudogene clinical application, 32 HNSCC tissues (16 oral squamous cell carcinoma tissues and 16 thyroid cancer tissues) from the Tumor Resource Bank of Sun Yat-sen University Cancer Center were used to verify the relative RNA expression of these 10 m^6^A-associated by qPCR. Results show oncogene pseudogenes (PDIA3P1, LDHAP4, LDHAP7, EEF1A1P6, and EEF1A1P11) displayed high expressions in the low-OS subtype and that tumor-suppressor pseudogenes (SDHAP1, SDHAP3, DDX12P, CLUHP3, and RRN3P3) demonstrated high expressions in the high-OS subtype (Figure 2C-D), which was consistent with our above results (Figure 2A).

Since the above screened 10 m^6^A-associated pseudogenes are an independent prognostic factor in HNSCC patients, we would expect to see a higher expression of oncogene pseudogenes in companions with worse survival outcomes, as well as higher expression of tumor-suppressor pseudogenes in companions with better survival outcomes. To test this, we examine the associations between the expression of m^6^A regulators and m^6^A-associated pseudogenes with overall survival in HNSCC patients from the TCGA dataset. We found that patients with high expression of three m^6^A regulators in TCGA dataset (Figure 2E) and nine m^6^A regulators in the GEO dataset (Figure S6A) had a significantly worse outcome than those with low expression, suggesting that high expression of m^6^A regulators might have accelerated the progression of the tumor. It's worth noting that *WTAP*, *YTHDC1*, and* YWHAG* represented significant differences both in TCGA and in the GEO dataset, implying the critical function of m^6^A regulators in HNSCC, in line with the pivotal roles of *YTHDC1* and *WTAP* in the previous findings (Figure 1E). Patients with high expression of oncogene pseudogenes had strikingly shorter survival than those with low expression (log-rank test; *PDIA3P1*, *P* = 0.046; *LDHAP4*, *P* = 0.004; *LDHAP7*, *P* = 0.014; *EEF1A1P6*, *P* = 0.025; *EEF1A1P11*, *P* = 0.007; Figure 2F), manifesting that high expression of oncogene pseudogenes might correlate with high malignancy of the tumor. In contrast, patients with high expression of tumor-suppressor pseudogenes had significantly better outcome than those with low expression (log-rank test; *SDHAP1*, *P* = 0.023; *SDHAP3*, *P* = 0.036; *DDX12P*, *P* = 0.007; *CLUHP3*, *P* = 0.017; *RRN3P3*, *P* = 0.014; Figure 2G), which explained that high expression of tumor-suppressor pseudogenes was associated with high benignancy of tumor. What's more, this conclusion has been further verified in the GEO dataset (Figure S6B-C).

To better show, the correlation between the expression of pseudogenes and the clinical outcome of patients, nomograms for evaluating the risk of HNSCC were developed for HNSCC patients based on risk factors identified by the multivariate logistic regression analysis. According to the results of the multivariate analysis, we included risk scores based on m^6^A-associated pseudogenes and some important clinical features to build a nomogram for predicting the 1-year, 3-year, and 5-year prognoses of HNSCC patients. Although our analysis did not identify Gender, Pathology_T_Stage, Pathology_M_Stage, Hpv_status, Laterality, and Histologic_grade as independent predictive factors, we took into account these variables in the nomogram. The total point was calculated according to the Oxford classification recommendations and principle, which was further transformed into probability (see the bottom scale) (Figure S7A). The calibration curves expressed a good consistency between the nomogram-predicted progression probability and the actual progression probability. And the calibration curves also showed our nomogram performing well in predicting the 1-year, 3-year, and 5-year prognosis of HNSC patients (Figure S7B). The above evidence strongly proved that expressions of 10 m^6^A-associated pseudogenes are significantly associated with survival outcomes of HNSCC patients.

### The expression pattern of m^6^A-associated prognostic pseudogenes was significantly correlated with antitumor immune response

**Figure 3:**
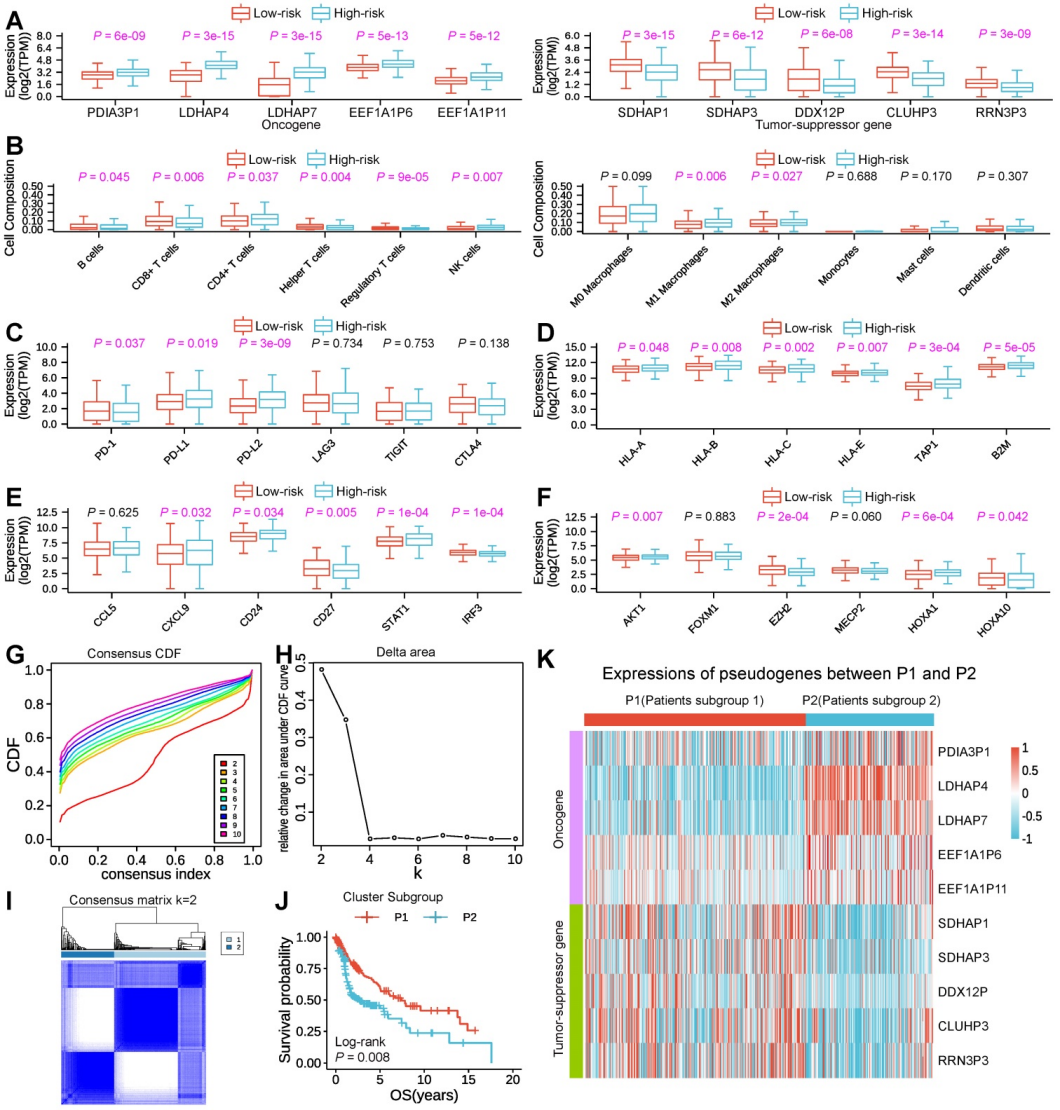
** Expression pattern of m^6^A-associated prognostic pseudogenes was significantly correlated with antitumor immune response. (A)** Boxplot revealing comparisons of expression levels of oncogenes (*PDIA3P1*, *LDHAP4*, *LDHAP7*, *EEF1A1P6*, *EEF1A1P11*) and tumor-suppressor genes (*SDHAP1*, *SDHAP3*, *DDX12P*, *CLUHP3*, *RRN3P3*) between low-risk and high-risk subtypes. **(B)** Boxplot showing comparisons of cell composition fraction of B cells, CD8+ T cells, CD4+ T cells, helper T cells, regulatory T cells, activated natural killer (NK) cells, M0 macrophages, M1 macrophages, M2 macrophages, monocytes, mast cells, and activated dendritic cells between low-risk and high-risk subtypes. **(C)** Boxplot displaying comparisons of expressions of *PD-1*, *PD-L1*, *PD-L2*, *LAG3*, *TIGIT*, and *CTLA4* between low-risk and high-risk subtypes. **(D)** Boxplot manifesting comparisons of expressions of *HLA*-*A*, *HLA*-*B*, *HLA*-*C*, *HLA*-*E*, *TAP1*, and *B2M* between low-risk and high-risk subtypes. **(E)** Boxplot comparing the expressions of *CCL5*, *CXCL9*, *CD24*, *CD27*, *STAT1*, and *IRF3* between low-risk and high-risk subtypes. **(F)** Boxplot comparing the expressions of kinase genes (*AKT1*, *FOXM1*, *E2F2*, *MECP2*, *HOXA1*, and *HOXA10*) between low-risk and high-risk subtypes. The *P*-value of comparisons between the two subtypes was calculated through the Wilcoxon test. Purple represents *P*-value < 0.05. **(G)** Plot of consensus clustering cumulative distribution function (CDF) for k = 2 to 10. **(H)** Line graph showing the relative change in area under CDF curve for k = 2 to 10. **(I)** The plot of consensus clustering of 500 HNSCC with k = 2, indicates P1/2 subgroups were identified by consensus clustering of the 10 m^6^A-associated pseudogenes in the TCGA dataset. **(J)** Kaplan-Meier survival curve reveals that the HNSCC patients in the P1 subgroup displayed significantly longer overall survival than those in the P2 subgroup (*P* = 0.008). **(K)** Heatmap displaying the expression pattern of m^6^A-associated pseudogenes between P1 and P2 subgroups.

To investigate and make clear the correlation between the expression pattern of m^6^A-associated pseudogenes and antitumor immune response in HNSCC, we compared the expressions of m^6^A-associated pseudogenes, estimated the immune cell infiltration through CIBERSORT, and assessed the expressions of T cell exhausted, antigen presentation, interferon activity, kinase, cytolytic and integrin genes in tumor tissues between low- and high-risk subtypes. The expression pattern of the 10 m^6^A-associated pseudogenes across low- and high-risk subtypes indicated that the low-risk subtype had lower expressions of oncogene pseudogenes as well as higher expressions of tumor-suppressor pseudogenes, while the high-risk subtype showed the opposite trends (Figure 3A). Studying the important role of immune-infiltrating cells in tumor immunity helps us to specify the best immunotherapy regimen. Through assessing the immune cell infiltration, we found that the low-risk subtype had a prominently higher number of tumor-infiltrating B cells, CD8^+^ T cells (as known as cytotoxic T cells), helper T cells, regulatory T cells, and a lower fraction of activated natural killer cells, M1 macrophage cells, M2 macrophage cells than high-risk (Figure 3B), suggesting an enhanced immunosurveillance in the low-risk subtype. Of note, CD4^+^ T cells, a renowned member of the helper T cell population, showed a significantly higher quantity in high-risk than in the low-risk subtype (Figure 3B). Evaluating the expression level of immunomodulatory genes in tumor immunity contributes to helping us to target specific immunotherapy targets. Then comparisons of expressions of immunomodulatory genes were conducted. In terms of T-cell exhausted genes, the low-risk subtype was dramatically associated with higher expressions of *PD*-*1* than the high-risk subtype (Figure 3C), which is a key gene of T-cell exhaustion markers. However, the high-risk subtype had remarkably higher expressions of *PD*-*L1* and *PD*-*L2*, which also account low-risk subtype having a longer Survival period (Figure 3C). Concerning antigen-presenting genes, our findings revealed that high-risk had significantly higher expressions of *HLA*-A, *HLA*-B, *HLA*-C, *HLA*-E, *TAP1*, and *B2M* than low-risk (Figure 3D), which can activate cytotoxic T cells. Regarding interferon activity genes, we surprisingly found that the expressions of CXCL9, *CD24*, and *STAT1* were also significantly higher in high-risk than the low-risk subtype, while the expressions of *CD27* and *IRF3* show the opposite trend (Figure 3E). In addition, the low-risk subtype was also associated with higher expressions of AKT1, *E2F2*, *MECP2*, *HOXA1*, and *HOXA10* (Figure 3F), several important regulatory genes for kinase activity. Besides, there is a significant difference in the expressions of cytolytic genes (*CYTH1*, *CYTH2*, *CYTH3*) and integrin genes (*ITGA* family genes and *ITGB* family genes) (Figure S8A-C) between low- with high-risk subtype.

In consideration of the large amounts of m^6^A-associated prognostic pseudogenes, we performed consensus clustering of the 10 m^6^A-associated prognostic pseudogenes through dimensionality reduction analysis in the subsequent study. Based on the similarity of pseudogenes expression, when clustering stability increased from k = 2 to 10, k = 2 seemed to be the optimal selection in the TCGA dataset (Figure 3G-I). Thus, we divided the 500 HNSCC patients into two subgroups by making 2 the k value, namely, P1 (Patients subgroup 1) and P2 (Patients subgroup 2). We found that patients in the P1 subgroup had a significantly better outcome than those in the P2 subgroup by Kaplan-Meier analysis (median overall survival 7.5 years vs. 3.5 years, log-rank test, *P* = 0.008, Figure 3J). And our results indicated that the P1 subgroup had higher expressions of tumor-suppressor pseudogenes and lower expressions of oncogene pseudogenes, while the P2 subgroup showed the contrary tendency (Figure 3K), which was highly consistent with the low-risk and high-risk subgroups. Specifically, compared with P2, the P1 subgroup had significantly higher expressions of 5 tumor-suppressor pseudogenes and significantly lower expressions of 5 oncogene pseudogenes (Figure S9A).

The correlation results between the expression pattern of m^6^A-associated pseudogenes and immune response across P1 and P2 subgroups were also consistent with the low-/high-risk subtype (Figure S9B-I). Besides, the P1 subgroup was significantly associated with few HPV infections (*P* = 0.009) and lower Pathologic M stage (*P* = 0.047) than P2 (Table S5). By comparing the difference between P1/P2 with the high/low-risk subtype, we found that the low-risk subtype contains 222 patients in the P1 and 28 patients in the P2 subgroup, and the high-risk subtype contains 95 patients in the P1 and 155 patients in the P2 subgroup. The P-value of the correlation between the low/high-risk subtype and Pathologic T stage, Pathologic M stage, and Vital status was more significant than the P1/P2 subgroup (Table S4 and S5). These observations were highly in line with those of the risk score mentioned above, which bolstered that the expression pattern of 10 m^6^A-associated pseudogenes was significantly associated with outcome in HNSCC.

To better elucidate the association between m^6^A-associated pseudogenes and tumor malignancy, we identified the differentially expressed genes between the P1 and P2 subgroups and annotated their functions. GO analyses indicated that upregulated genes in P1 were strikingly enriched in tumor-related biological processes (Figure S10A), including STAT pathway and natural killer cell activation, etc. GO analysis also revealed that downregulated genes in P1 were enriched in the cellular process involved in reproduction in the multicellular organism and so on (Figure S10B). Furthermore, “AKT_UP_MTOR_DN.V1_UP”, “MTOR_UP.N4.V1_DN” and “GO_SOMATIC_DIVERSIFICATION_OF_IMMUNE_RECEPTORS_VIA_SOMATIC_MUTATION” were significantly enriched in the P1 subgroup indicated by GSEA (Figure S10C). All these results indicate that the expression pattern of m^6^A-associated pseudogenes was closely associated with the malignancy of HNSCC.

Our findings partially explained the above discovery that tumors in the low-risk and P1 subgroups had stronger immunogenicity and thus presented a higher fraction of active immune cell infiltrations. Therefore, convergent evidence supported that m^6^A-associated prognostic pseudogenes played an important role in the antitumor response, which might serve as potential biomarkers for immunotherapy.

### m^6^A-associated pseudogenes can regulate targeted immune-involved genes via miRNAs

**Figure 4:**
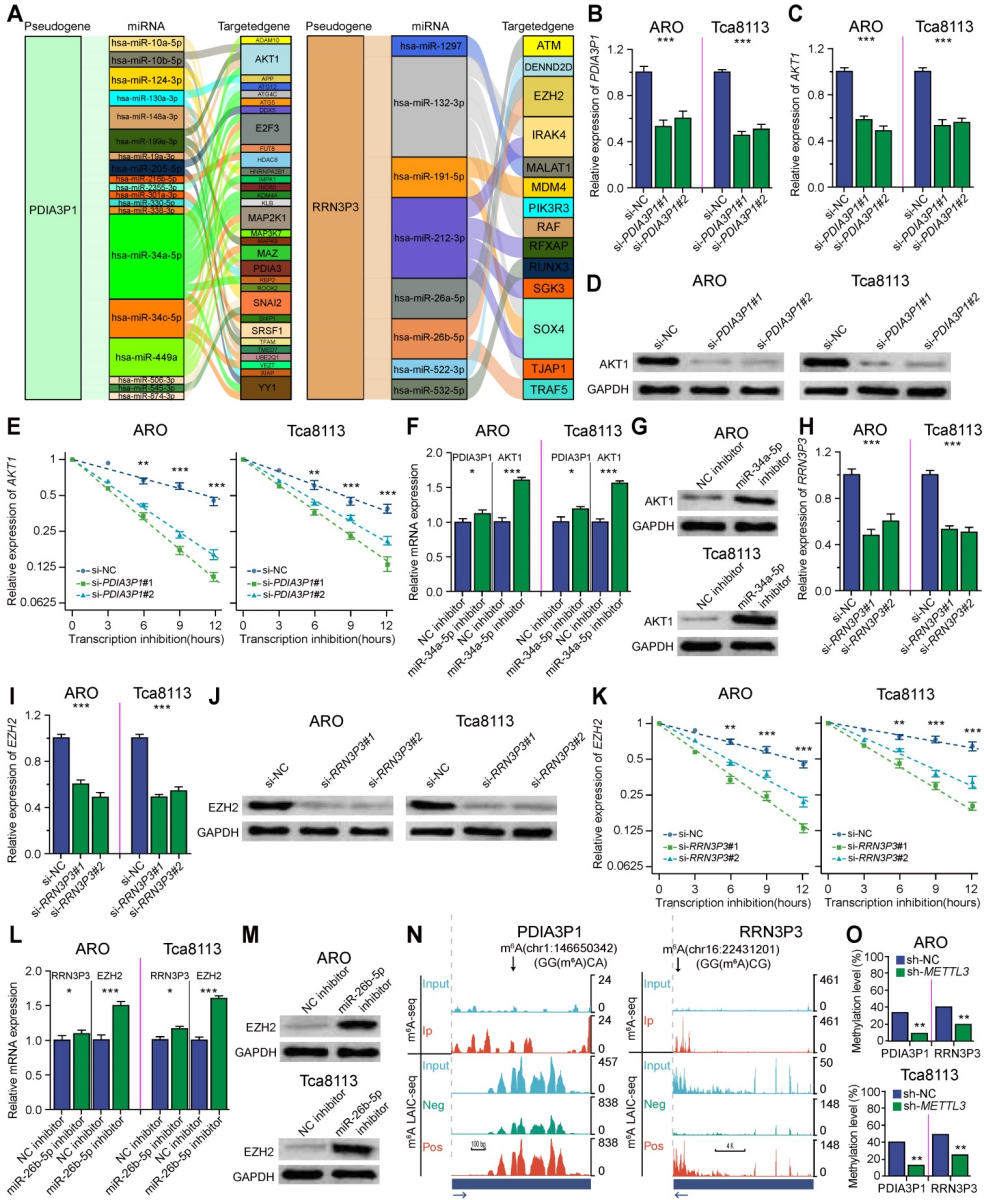
** m^6^A-associated pseudogene can regulate targeted immune-involved genes via miRNAs. (A)** Sankey showing pseudogenes together with binding miRNAs and target genes with | *R* | ≥ 0.3 and *P* < 0.05 were used to construct the pseudogene-miRNA-target gene regulatory networks by subtypes of oncogene pseudogene *PDIA3P1* and tumor-suppressor pseudogene *RRN3P3*. The column on the left represented pseudogenes, which are located at the cores of the networks. The column in the middle and the column on the right stand for binding miRNAs and target genes, respectively. **(B-G)** Experimental validation of *PDIA3P1* affects the expression of *AKT1* via miR-34a-5p in ARO and Tca8113 cell lines. **(B)** Relative gene expression of *PDIA3P1* after *PDIA3P1* knockdown using siRNA. **(C)** Relative gene expression of *AKT1* after *PDIA3P1* knockdown using siRNA. **(D)** Western blot comparing the protein levels of AKT1 in control and *PDIA3P1* knockdown cells. **(E)** The relative expression of *AKT1* at different time points after transcription inhibition in control and *PDIA3P1* knockdown cells respectively. Error bars represent standard errors. **(F)** The relative expression of *PDIA3P1* and *AKT1* after adding control inhibitor versus miR-34a-5p inhibitor. **(G)** Western blot comparing the protein levels of AKT1 after adding control inhibitor versus miR-34a-5p inhibitor. (H-M) Experimental validation of *RRN3P3* affects the expression of *EZH2* via miR-26b-5p in ARO and Tca8113 cell lines. **(H)** Relative gene expression of *RRN3P3* after *RRN3P3* knockdown using siRNA. **(I)** Relative gene expression of *EZH2* after *RRN3P3* knockdown using siRNA. **(J)** The relative expression of *EZH2* at different time points after transcription inhibition in control and *RRN3P3* knockdown cells respectively. Error bars represent standard errors. **(K)** Western blot comparing the protein levels of EZH2 in control and *RRN3P3* knockdown cells. **(L)** The relative expression of *RRN3P3* and *EZH2* after adding control inhibitor versus miR-26b-5p inhibitor. **(M)** Western blot comparing the protein levels of EZH2 after adding control inhibitor versus miR-26b-5p inhibitor. **(N)** UCSC genome browser tracks m^6^A-seq and m^6^A-LAIC-seq data indicating m^6^A peaks and m^6^A levels of oncogene pseudogene *PDIA3P1* and tumor-suppressor pseudogene *RRN3P3*. Read-coverage tracks of input, m^6^A-negative, and m^6^A-positive fractions of m^6^A-LAIC-seq shown along with overlay tracks of m^6^A-seq (cyan for input and red for RIP; predicted m^6^A sites in m^6^A peaks are indicated by arrows). Read coverage (y-axis) of m^6^A negative and m^6^A positive are normalized as previously described 17 to reflect the calculated m^6^A levels (i.e., equal signals in m^6^A positive (eluate) versus m^6^A negative (supernatant) suggest m^6^A levels of 50%), while input and IP tracks of m^6^A-seq are shown for optimal viewing at the top panel. * *P*< 0.05; ** *P*< 0.01; *** *P*< 0.001 (two-tailed t-test). **(O)** The m^6^A methylation level of the pseudogenes at specific modification sites (Chr1:146650342, GG(m^6^A)CA on *PDIA3P1*; Chr16:22431201, GG(m^6^A)CG on *RRN3P3*) using SELECT in control and *METTL3* knockdown ARO and Tca8113 cell.

To illuminate the potential mechanism of how m^6^A-associated pseudogenes regulated anti-tumor immune response, we constructed a pseudogene-miRNA-targeted immune-involved gene regulatory network. Underlying miRNAs binding to the 10 pseudogenes were identified using the dreamBase and miRNA target genes were extracted by the miRTarBase, which were verified by at least two strong experiments (Table S6). We calculated expression correlations between each pseudogene and its miRNA target genes using Pearson correlation analysis. Target genes with | *R* | ≥ 0.3 and *P* < 0.05 were picked up (Table S7). Ultimately, 4 tumor-suppressor pseudogenes (*SDHAP1*, *SDHAP3*, *DDX12P*, *RRN3P3*) together with 26 microRNAs and 138 targeted genes, and 4 oncogene pseudogenes (*PDIA3P1*, *LDHAP7*, *EEF1A1P6*, *EEF1A1P11*) together with 28 microRNAs and 58 targeted genes, were used to build the pseudogene-miRNA-target gene regulatory networks and visualized using Sankey diagram (Figure 4A; Figure S11A-F). Oncogene pseudogene *PDIA3P1*, acting as a decoy of hsa-miR-34a-5p, hsa-miR-10b-5p, hsa-miR-199a-3p, and hsa-miR-19a-3p downregulated the expression of *AKT1* and then downregulated the infiltrations of some immune cells (including CD4+ T and M1 macrophages cells) through calcium signaling and signal transduction (Figure 4A, left panel) in the low-risk subtype. Tumor-suppressor pseudogene *RRN3P3* upregulated the expression of *EZH2* by competitively binding hsa-miR-26a-5p and hsa-miR-26b-5p, which explained the higher expression of *EZH2* in the low-risk subtype (Figure 4A, right panel). Other pseudogenes also played regulatory roles in signaling pathways coving oncogenic transformation, cell proliferation, and cell migration as ceRNAs (Figure S11A-F). The pseudogene-miRNA-targeted immune-involved gene regulatory networks partially clarified the mechanism of how pseudogenes were involved in regulating the immune response in HNSCC.

To experimentally test whether the m^6^A-associated pseudogenes affect the expression of their targeted immune-involved genes, we selected representative oncogene pseudogene *PDIA3P1* and tumor-suppressor pseudogene *RRN3P3* for further validation in ARO and Tca8113 cell lines, which were two representatives HNSCC cell lines. First, we tested whether oncogene pseudogene* PDIA3P1* can regulate its targeted *AKT1*. We found knockdown of *PDIA3P1* using siRNA significantly down-regulated gene expression as well as the protein expression of AKT1 (Figure 4B-D), consistent with our observation that their gene expressions were positively correlated (Figure 3A and 3F). To test whether *PDIA3P1* modules *AKT1 via* ceRNA mechanism, we first verified that knockdown of *PDIA3P1* was able to promote the degradation of *AKT1* (Figure 4E). Then we selected miR-34a-5p for experimental validation. Our findings revealed that inhibition of miR-34a-5p significantly increased the mRNA and protein expression of both *PDIA3P1* and *AKT1*, indicating that miR-34a-5p targets both *PDIA3P1* and *AKT1* (Figure 4F-G). Next, we tested whether Tumor-suppressor pseudogene *RRN3P3* can regulate its targeted *EZH2*. We found knockdown of *RRN3P3* using siRNA significantly down-regulated gene expression as well as the protein expression of EZH2 (Figure 4H-J), in line with our observation that their gene expressions were positively correlated (Figure 3A and 3F). Then to test whether *RRN3P3* modules *EZH2 via* ceRNA mechanism, we first confirmed that knockdown of *RRN3P3* could promote the degradation of *EZH2* (Figure 4K). Then we selected miR-26b-5p for experimental validation. We found inhibition of miR-26b-5p significantly increased the mRNA and protein expression of both *RRN3P3* and *EZH2*, indicating that miR-26b-5p targets both *RRN3P3* and *EZH2* (Figure 4L and 4M).

To better study the function of m^6^A-associated pseudogenes, we utilized m^6^A-LAIC-seq to quantify the m^6^A levels on pseudogenes on a transcriptome-wide scale and used m^6^A seq technology to identify m^6^A peaks and m^6^A sites on pseudogenes. Here, we took advantage of our previously published m^6^A levels of pseudogenes 20, which were calculated from m^6^A-LAIC-seq data 17, to study the m^6^A levels of pseudogenes. And we used our previously published m^6^A peaks of pseudogenes, which were calculated from the m^6^A-seq data 21, to test whether the elevation of m^6^A levels on pseudogenes was due to *de novo* formation of m^6^A peaks on pseudogenes or promoted methylation on ancestral m^6^A sites. As shown in Figure 4N, m^6^A-seq identified two m^6^A peaks on oncogene pseudogene *PDIA3P1* (left panel) and one m^6^A peak on tumor-suppressor pseudogene *RRN3P3* (right panel), and the m^6^A-LAIC-seq data showed that full-length RNAs of pseudogenes were significantly enriched in m^6^A positive fraction. Similar results were also observed for other m^6^A-associated pseudogenes. The above results suggest that the m^6^A levels of m^6^A-associated pseudogenes are increased attributed to the *de novo* formation of m^6^A sites. Our previous discovery revealed that m^6^A on processed pseudogenes plays a novel evolutionary role in removing the unnecessary processed pseudogenes to mitigate their interference in the expression of protein-coding genes 20. To determine the m^6^A methylation levels at each site on pseudogenes, we used absolute quantification in the m^6^A modification assay, which is a new and ultrasensitive quantitation assay for the accurate determination of m^6^A at single-nucleotide resolution. We found that METTL3 could significantly methylate *PDIA3P1* mRNA on the Chr1:146650342 site and* RRN3P3* mRNA on the Chr16:22431201 site (Figure 4O).

### HNSCC patients in the high-risk subtype could benefit more from immune checkpoint inhibitors therapy

**Figure 5:**
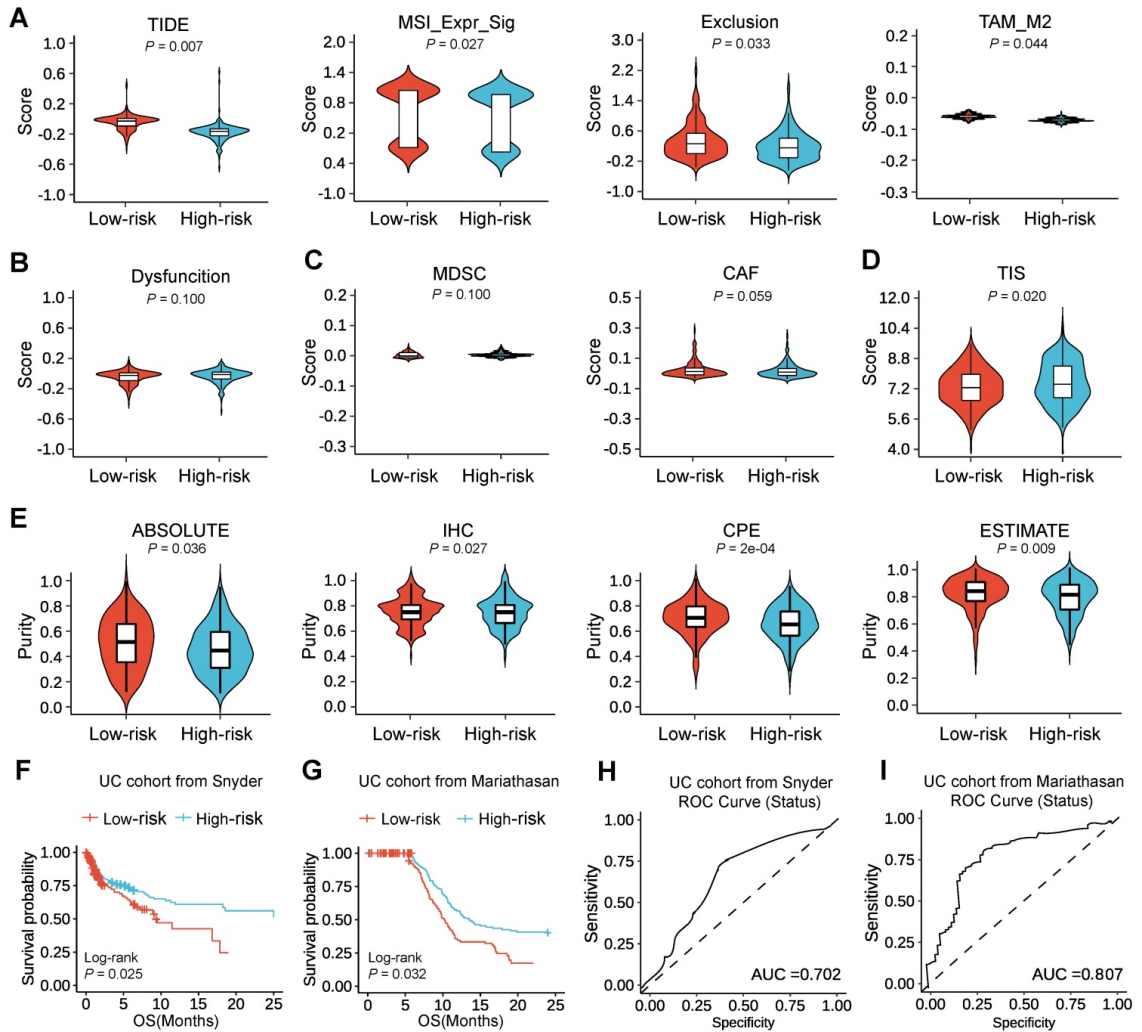
** HNSCC patients in the high-risk subtype could benefit more from immune checkpoint inhibitors therapy. (A)** Violin illustration showing comparisons of TIDE, MSI_Expr_Sig, Exclusion, and TAM_M2 values in different low-/high-risk subtypes, which represent TIDE, MSI, T cell exclusion dysfunction, and TAM score respectively. **(B)** Violin illustration comparing dysfunction value in different low-/high-risk subtypes, which stand for T cell dysfunction score. **(C)** Violin illustration comparing MDSC and CAF values in different low-/high-risk subtypes, which stand for MDSC and CAF scores respectively. **(D)** Violin illustration comparing TIS score in different low-/high-risk subtypes, which stand for TIS score. **(E)** Violin illustration indicating comparisons of purity calculated by four methods (ABSOLUTE, IHC, CPE, and ESTIMATE) in different low-/high-risk subtypes. The *P*-value of comparisons between the two subtypes was calculated through the Wilcoxon test. **(F)** Kaplan-Meier survival curve revealed that the UC patients from the Snyder cohort in the low-risk subtype displayed significantly longer overall survival than those in the high-risk subtype (*P* = 0.025). **(G)** Kaplan-Meier survival curve revealed that the UC patients from the Mariathasan cohort in the low-risk subtype displayed significantly longer overall survival than those in the high-risk subtype (*P* = 0.032). **(H)** The ROC curve shows AUC for the predictive value of m^6^A-associated pseudogene in the UC cohort from Snyder. **(I)** The ROC curve shows AUC for the predictive value of m^6^A-associated pseudogene in the UC cohort from Mariathasan.

To elucidate the benefits situation of ICI therapy in different low-/high-risk subtypes, we then used tumor immune dysfunction and exclusion (TIDE) to evaluate the potential clinical efficacy of immunotherapy in different low-/high-risk subtypes. A higher TIDE prediction score indicated a higher potential for immune evasion, which represented that the patients were less likely to benefit from ICI therapy. Here, we found that the high-risk subtype had a lower TIDE score than the low-risk subtype, suggesting that high-risk patients could benefit more from ICI therapy than low-risk patients (Figure 5A). Since a higher TIDE prediction score was associated with a worse outcome, the high-risk subtype with a low TIDE score might have a better prognosis than the low-risk subtype with a high TIDE score from immune checkpoint inhibitors therapy, which was consistent with that PD-L1 expression was positively correlated with the efficacy of immune checkpoint inhibitors. Moreover, we found that the low-risk subtype had a higher microsatellite instability (MSI) score, T cell exclusion, and tumor-associated macrophages M2 (TAM_M2) (Figure 5A), but there was no difference in T cell dysfunction (Figure 5B), myeloid-derived suppressor cell (MDSC) and cancer-associated fibroblasts (CAF) (Figure 5C) between the two subtypes. In addition, the tumor inflammation signature (TIS) score based on the 18 signature genes (List S6) was calculated as an average value of the log2-scale normalized expression. And we also found that the high-risk subtype with a high TIS score than the low-risk subtype with a low TIS score, which suggested that the high-risk subtype might have a better outcome by estimating the TIS score (Figure 5D; Table S8). Besides, we evaluated the purity of the tumor through several methods including ABSOLUTE, IHC, CPE, and ESTIMATE, convergently suggesting that the low-risk subtype might have a higher purity than the high-risk subtype (Figure 5E), indicating a higher percentage of cancer cells, which might explain the reasons for the worse prognosis of patients in low-risk subtype after ICI therapy.

Since the high-risk subtype was able to benefit more from ICI therapy than the low-risk subtype, we would expect to see the high-risk subtype in companion with better survival outcomes after the treatment of ICI than the low-risk subtype. To test this, we assessed the prognostic value of m^6^A-associated pseudogenes in two urothelial cancer (UC) cohorts with anti-PD-L1 therapy 22, 23. Surprisingly, we could find that high-risk patients had better OS than low-risk patients (Figure 5F-G). We could also find that the performance of m^6^A-associated pseudogenes was consistent in the UC cohort from Snyder 22 and the UC cohort from Mariathasan 23, at 12 months of follow-up (Figure 5H-I). Thus our study indicated that the predictive value of the expression pattern of m^6^A-associated pseudogenes was comparable with TIDE and TIS for OS.

### Identification and experimental validation of potential therapeutic agents with higher drug sensitivity for HNSCC patients in the high-risk subtype

In addition to HNSCC patients with m^6^A-associated pseudogene expression patterns who may benefit from immunotherapy, we also expect these patients to benefit from therapeutic agents. To solve this issue, we took advantage of two powerful datasets, which could quickly screen thousands of drugs from hundreds of human cancer models on an unprecedented scale. Specifically, the Cancer Therapeutics Response Portal (CTRP) and Profiling Relative Inhibition Simultaneously in Mixtures (PRISM) datasets, which contain the gene expression profiling and drug sensitivity profiling of hundreds of cancer cell lines (CCLs), can be used to build a prediction model of drug response. 160 of these compounds are shared between the two datasets. The CTRP has unique 322 compounds and the PRISM has unique 1288 compounds. Results showed that there were 1770 compounds both in the CTRP and PRISM datasets after removing duplication (Figure S12A). Compounds with NAs in more than 20% of the samples were excluded. Ultimately, 654 CCLs with 354 compounds in the CTRP dataset, as well as 439 CCLs with 1291 compounds in the PRISM dataset were utilized for subsequent analysis.

**Figure 6:**
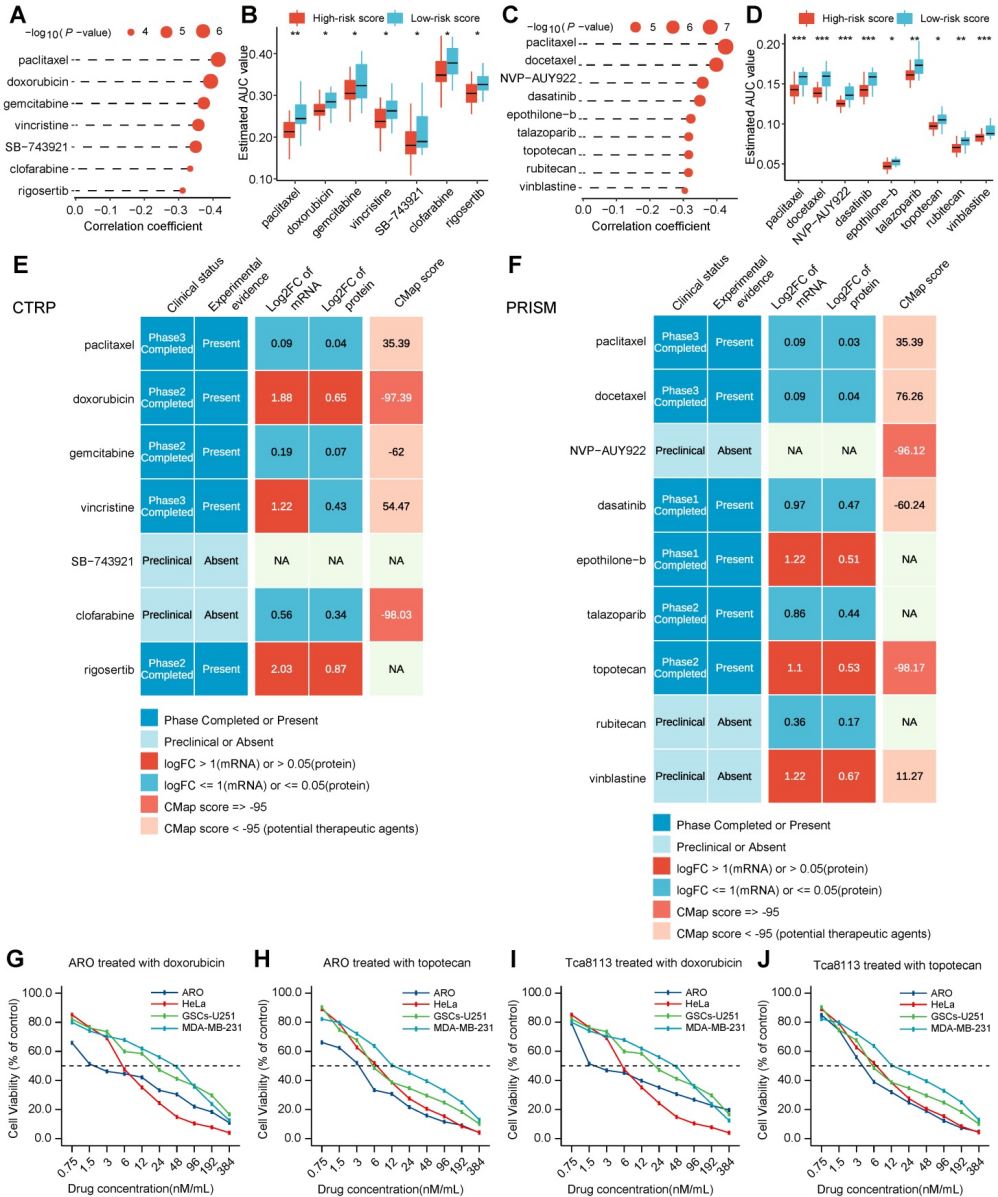
** Identification and experimental validation of potential therapeutic agents with higher drug sensitivity for HNSCC patients in the high-risk subtype. (A)** Horizontal column diagram showing the correlation coefficient and *P*-value of Spearman's correlation analysis of seven CTRP-derived compounds. **(B)** Boxplots displaying estimated AUC value of differential drug response analysis of seven CTRP-derived compounds between high-risk score patients with low-risk score patients. Note that lower values on the y-axis of boxplots imply greater drug sensitivity. The value between the two subtypes was compared through the Wilcoxon test. ns denotes no significance, * denotes *P* < 0.05, ** denotes *P* < 0.01, *** denotes *P* < 0.001 and **** denotes *P* < 0.0001. **(C)** Horizontal column diagram showing the correlation coefficient and *P*-value of Spearman's correlation analysis of nine PRISM-derived compounds. **(D)** Boxplots displaying estimated AUC value of differential drug response analysis of nine PRISM-derived compounds between high-risk score patients with low-risk score patients. Note that lower values on the y-axis of boxplots imply greater drug sensitivity. The value between the two subtypes was compared through the Wilcoxon test. ns denotes no significance, * denotes *P* < 0.05, ** denotes *P* < 0.01, *** denotes *P* < 0.001 and **** denotes *P* < 0.0001. **(E)** Identification of most promising therapeutic CTRP-derived agents for high-risk score patients according to the evidence from multiple sources. Combined heatmap showing seven CTRP-derived agents. mRNA or protein expression was compared by fold change (FC) differences of drug targets between tumor and normal tissue (FC >0 represents up-regulated in tumor tissue). **(F)** Identification of most promising therapeutic PRISM-derived agents for high-risk score patients according to the evidence from multiple sources. Combined heatmap showing nine PRISM-derived agents. mRNA or protein expression was compared by fold change (FC) differences of drug targets between tumor and normal tissue (FC >0 represents up-regulated in tumor tissue). **(G-J)** MTT assay to determine the IC_50_ value of the different drugs (doxorubicin and topotecan) and analyze their effect on head and neck cancer cell lines (ARO and Tca8113) and other sensitive cell lines (HeLa, GSCs-U251, and MDA-MB-231) viability. There are three biological replicates of cell viability per drug concentration. Line graph showing the different drug concentrations used and the corresponding cell viability between ARO with other cells treated with doxorubicin (G), between ARO with other cells treated with topotecan (H), between Tca8113 with other cells treated with doxorubicin (I) and between Tca8113 with other cells treated with topotecan (J). The IC_50_ values of ARO cells for doxorubicin and topotecan were 1.6 nM, and 3.5 nM, respectively. The IC_50_ values of Tca8113 cells for doxorubicin and topotecan were 2.2 nM, and 4.6 nM, respectively.

To identify potential therapeutic agents in HNSCC patients, two different methods were used to confirm candidate drugs with higher drug sensitivity in high-risk score patients (Figure S12B). The analyses were conducted using CTRP and PRISM datasets, respectively. Firstly, differential drug response approaches between high-risk score (top decile) and low-risk score (bottom decile) groups were adopted to identify compounds with lower calculated AUC values in the high-risk score group with log2FC > 0.10 (Table S9; Table S10). Secondly, Spearman correlation calculation between AUC value and risk score was performed to screen compounds through a negative correlation coefficient (Spearman's *R* < -0.30 for both CTRP and PRISM). The above analyses yielded seven CTRP-derived compounds (including paclitaxel, doxorubicin, gemcitabine, vincristine, SB-743921, clofarabine, and rigosertib) (Figure 6A-B) and nine PRISM-derived compounds (including paclitaxel, docetaxel, NVP-AUY922, dasatinib, epothilone-b, talazoparib, topotecan, rubitecan, and vinblastine) (Figure 6C-D). These screened compounds had lower calculated AUC values in the high-risk score subtype, as well as a negative correlation with a risk score for CTRP (Figure 6B) and PRISM datasets (Figure 6D), which might be potential therapeutic agents in HNSCC patients with a high-risk score.

Despite the 16 candidate therapeutic agents identified showing a higher drug sensitivity in high-risk score HNSCC patients, the above screening method alone was not able to support the conclusion that these agents had a therapeutic effect on HNSCC. Thus, multiple perspective analyses were subsequently performed to investigate the therapeutic potential of these compounds in HNSCC. Firstly, fold-change differences in the expression levels (including mRNA-level and protein-level) of candidates' drug targets between tumor and normal tissue were calculated. In the calculation results, a higher fold-change value manifested a greater potential and better efficacy of the candidate compound for HNSCC treatment (Table S11). Secondly, the CMap analysis approach was adopted to identify compounds in which gene expression patterns were contrary to the HNSCC-specific expression patterns (in other words, gene expression increased in tumor tissues but decreased by treatment of certain agents). Four compounds, including doxorubicin, clofarabine, NVP-AUY922, and topotecan, had CMap scores < -95 (Table S12), on behalf, that these compounds might have a potential therapeutic effect on HNSCC (Figure 6E-F). Thirdly, comprehensive analyses including drug data query in DrugBank and literature search in PubMed were conducted to look for the experimental validation and clinical evidence of candidate agents in treating HNSCC (Figure 6E-F). The above three results were presented on the middle, right, and left of the panel respectively (Figure 6E-F). Finally, doxorubicin, and topotecan, which had robust multi-level evidence including *in vitro* and in silico, were deemed to hold the most promising therapeutic potential in HNSCC patients with a high-risk score.

To experimentally test whether potential therapeutic agents with higher drug sensitivity for HNSCC patients in high-risk subtype affect the tumor cell viability, we performed IC_50_ (in other words, the concentration of drug which causes 50% cell viability) assay of doxorubicin and topotecan in head and neck cell lines (ARO and Tca8113) and other corresponding sensitive cell lines (HeLa, GSCs-U251, and MDA-MB-231). The effect of doxorubicin and topotecan on cell viability after 24 h treatment was evaluated using a colorimetric MTT assay in both cell lines. The two therapeutic agents have their distinct effects on cell viability at 10 different concentrations. Our findings revealed that the cell survival rate was observed to generally decrease with an increase in drug concentration, suggesting a dose-dependent behavior. The IC_50_ value of ARO cells for doxorubicin and topotecan were 1.6 nM, and 3.5 nM, respectively (Figure 6G-H). And the IC_50_ value of Tca8113 cells for doxorubicin and topotecan were 2.2 nM, and 4.6 nM, respectively (Figure 6I-J). The IC_50_ value of doxorubicin and topotecan for ARO and Tca8113 cells were less than those for HeLa, GSCs-U251, and MDA-MB-231 cells, as well as in previous studies, further verifying that HNSCC patients in the high-risk subtype could benefit more from doxorubicin and topotecan.

## Discussion

In this study, 10 m^6^A-associated pseudogenes were confirmed as promising prognostic indicators for HNSCC by a comprehensive analytical framework and classified into oncogene pseudogenes (*PDIA3P1*, *LDHAP4*, *LDHAP7*, *EEF1A1P6*, *EEF1A1P11*) and tumor-suppressor pseudogenes (*SDHAP1*, *SDHAP3*, *DDX12P*, *CLUHP3*, *RRN3P3*) owing to their different effects in prognosis in TCGA dataset. Then a risk score model based on the 10 m^6^A-associated pseudogenes was constructed s and found very good in predicting clinical outcomes in HNSCC, which was further validated in the GEO dataset, as well as our clinical tissues. More importantly, we found that the expression pattern of these 10 pseudogenes was dramatically associated with the immune response in terms of some aspects. Then, m^6^A-associated pseudogene-miRNA-targeted immune-involved gene regulatory networks were further performed to elucidate the underlying mechanisms that pseudogenes with m^6^A RNA modification could regulate antitumor immune response via miRNAs. Up to now, this is the first research to systemically clarify the prognostic value of m^6^A-associated pseudogenes and their regulatory roles in the host antitumor immune response of HNSCC. The novel discovery in this study also unveiled that the therapy of immune checkpoint inhibitors and doxorubicin and topotecan could enable HNSCC patients with the specific expression pattern of m^6^A-associated pseudogene to obtain good therapeutic effects.

Pseudogenes, as non-coding RNAs, are prevalently transcribed in the genome 24 and are nonfunctional and deleterious. A large proportion of unprocessed and processed pseudogenes can be removed in time in the RNA surveillance system. For example, most unprocessed pseudogenes can be degraded by Nonsense Mediated Decay (NMD) 25, and major processed pseudogenes can be cleaned by m^6^A on the RNAs of processed pseudogenes in our previously published articles 20. However, there are still some pseudogenes that have been nonremoved and still highly expressed, which can play increasingly important regulatory roles in diverse human diseases 26 as well as contain miRNA-binding elements and therefore increase their parental and other targeted genes by acting as ceRNA 27. Our results revealed that 6218 out of 13931 pseudogenes were expressed in TCGA HNSCC, which were our following research targets. So what role do these expressed pseudogenes play in cancer? A series of previous studies have depicted the crucial roles of pseudogenes in tumorigenesis and tumor development. For instance, *PTENP1* could suppress the progression of clear-cell renal cell carcinoma by acting as a ceRNA 28. *PKMP3*, *AC027612*.4, *HILS1*, *RP5*-*1132H15*.3, and *HSPB1P1* were found as prognostic predictors for lower-grade gliomas 29. *ANXA2P2*, *EEF1A1P9*, *FER1L4*, *HILS1*, and *RAET1K* were identified to be dramatically correlated with the survival of glioma 30. *RNA5SP141* could strongly enhance the RIG-I-mediated antiviral immune response to herpes simplex virus 1 31. However, there has been no focus on the role of pseudogenes in HNSCC, and our research will focus on this question.

m^6^A RNA modification is installed by m^6^A methyltransferases METTL3 and uninstalled by m^6^A demethylases FTO and ALKBH5 14, 15. A variety of RNA binding proteins can modulate diverse post-transcriptional processes of host mRNAs and non-coding RNAs by reading different m^6^A on these RNAs 14, 15, such as facilitating the cytosol degradation 13 and accelerating nuclear export of mRNAs 32. Increasing critical roles of m^6^A have been reported in many kinds of physiological and pathological processes of various cancers 33, including bladder cancer 35, gastric cancer 33, acute myeloid leukemia 33, and our published breast cancer 18. However, recent research mainly focuses on the function of m^6^A in mRNA, there are very few studies on the roles of m^6^A on pseudogenes. We firstly screened 842 pseudogenes by Pearson correlation analysis, and ultimately screened 10 pseudogenes by univariate and multivariate Cox proportional hazard regression. Exploring the role of these 10 pseudogenes, defined as m^6^A-associated prognostic pseudogenes, in HNSCC will be the main line of our next research. So far, two recent articles pointed out that the m^6^A-modified pseudogene *HSPA7* could be a new immunotherapy target for GBM patients 34, and aberrant m^6^A modification of the pseudogene *WTAPP1* results in increased translation of its protein-coding counterpart to promote pancreatic cancer progression 35. However, there has been no focus on the role of m^6^A on pseudogenes in HNSCC. Our findings revealed the prognostic function of screened 10 m^6^A-associated pseudogenes in HNSCC patients. To investigate whether 10 m^6^A-associated pseudogenes are an independent prognostic factor, we analyze the prognostic effects of 10 pseudogenes both in the TCGA and GEO datasets. Convergent evidence supports the 10 m^6^A-associated pseudogenes as an independent prognostic factor. To further demonstrate the prospect of m^6^A-associated pseudogene clinical application, we verified the correlation of pseudogene expression with clinical overall survival in a relatively small cohort including 32 HNSCC tissues from our hospital. Unfortunately, pseudogenes are non-coding proteins, so pseudogenes cannot express proteins, so we cannot verify the expression of pseudogenes at the protein level, which limits its potential clinical application value.

Our current study indicated that 10 m^6^A-associated pseudogenes were identified as promising prognostic indicators for HNSCC. Curiously, the oncogene pseudogenes happened to be processed pseudogenes, however, the tumor-suppressor pseudogenes were unprocessed pseudogenes, which was worthy of further study. Carcinogenic roles of *PDIA3P1* in HNSCC were in close agreement with that a higher expression of *PDIA3P1* was closely associated with a poorer recurrence-free survival of human hepatocellular carcinoma 36. The anticancer effect of *SDHAP1* was accordant with that *SDHAP1* upregulated *EIF4G2* level by sponging miR-4465 and therefore promoted the PTX-induced apoptosis in ovarian cancer 37. *SDHAP3* plays an important role in carcinogenesis in our study, which also displays strong involvement in neurodevelopmental disorders, and cancer susceptibility 38. The role of the remaining 7 m^6^A-associated pseudogenes (*LDHAP4*, *LDHAP7*, *EEF1A1P6*, *EEF1A1P11*, *DDX12P*, *CLUHP3*, and *RRN3P3*) has not been reported in the previous literature and has been coming up in our research, which was indispensable and identified as a good prediction of outcome in HNSCC. In summary, our study provides prospective prognostic predictors for HNSCC, which can better fulfill the principle of precise medicine.

Our findings firstly revealed that the expression pattern of the 10 m^6^A-associated pseudogenes was dramatically associated with tumor-infiltrating B cells, CD8^+^ T cells, helper T cells, and regulatory T cells, as well as the expressions of T cell exhausted markers including *PD*-*1*, *PD-L1*, *PD*-*L2*, *LAG3*, *TIGIT*, and *CTLA4*. What's more, antigen presentation genes, interferon activity genes, cytolytic genes, integrin genes, and kinase genes were also significantly associated with pseudogenes levels. To explain the mechanism of how m^6^A-associated pseudogenes regulated immune response, pseudogene-miRNA-targeted immune-involved gene regulatory networks were further constructed and validated experimentally to explain the underlying mechanisms and demonstrated that m^6^A-associated pseudogene can regulate antitumor immune-involved target genes via plenty of miRNAs. We found that the oncogene PDIA3P1, and tumor-suppressor RRN3P3, promote the RNA and protein expression of their targeted immune-involved genes AKT1 and EZH2 via miR-34a-5p and miR-26b-5p, respectively. Existing research indicates that AKT inhibition reduces PD-L1 expression in tumor cells, enhances activation and tumor infiltration of CD8+ T cells, and reduces tumor growth, accompanied by prolonged mouse survival 39. The previous study has shown that suppressing EZH2 activity resulted in increased numbers of myeloid-derived suppressor cells (MDSC) and fewer CD4+ and IFNγ+CD8+ T cells, which are involved in antitumor immunity 40. However, another study suggests that cell cycle-related kinase (CCRK) activated the EZH2/NF-κB/IL-6 cascade, which lead to the accumulation of polymorphonuclear MDSCs, downregulated PD-L1 expression, and decreased intratumoral CD8+ T cells 41. Inconsistent conclusions from two research teams further reveal the complexity of EAH2's role in tumors. What's more, we quantified the m^6^A levels and identified m^6^A peaks and m^6^A sites on m^6^A-associated pseudogenes, and verified the m^6^A level of the pseudogenes at specific modification sites in HNSCC cells by m^6^A-qPCR.

Our current study also firstly suggested the clinical application of m^6^A-associated pseudogenes in HNSCC, which can effectively predict treatment outcomes of immune therapy and drug therapy. By integrating with public datasets about immune therapy and drug sensitivity of CCLs, HNSCC patients in the high-risk subtype could benefit more from immune checkpoint inhibitors and promising potential therapeutic agents including doxorubicin and topotecan. Our study indicates that the high-risk subtype with higher PD-L1 expression, fewer infiltrating CD8+ T cells, and Low-MSI might have a better response from ICI treatment, which was possibly attributed to the effects of PD-L1 on apoptosis of CD8+ T cells 42. Our results of drug targets and compounds complemented each other, indicating a comprehensive view of a potential personalized treatment strategy, were further confirmed *in vitro* experiments. Overall, our study indicated that m^6^A-associated prognostic pseudogenes have not only provided new insights into personalized prognostication approaches but also thrown light on integrating tailored risk stratification with precision therapy.

## Conclusions

Our discovery revealed that the 10 m^6^A-associated prognostic pseudogenes significantly contribute to predicting immunotherapy benefits and therapeutic agents, which might bring some potential implications for both immunotherapy strategy and medical treatment in HNSCC. It provided new insights into personalized prognostication approaches and precision therapy.

## Materials and methods

### Data sources

The list of pseudogenes were compiled from GENCODE (https://www.encodeproject.org/) 43, Vega (http://vega.archive.ensembl.org/index.html), and Pseudogene.org databases (http://pseudogene.org/) 44. The gene expression profiles and corresponding detailed clinical information of HNSCC were downloaded from the TCGA data portal (http://firebrowse.org/) and GEO dataset (https://www.ncbi.nlm.nih.gov/geo/) by access number (GSE65858). Altogether, 544 samples (including 500 tumor tissues and 44 normal tissues) from TCGA and 270 tumor samples from GEO with pseudogene expression profiling and corresponding clinical data were included. The immune cell fraction data were obtained through CIBERSORT (https://cibersort.stanford.edu/) 45, 46. The antigen-presenting genes and immunomodulatory genes were obtained from TCIA (https://tcia.at/home) 47. The miRNAs binding to pseudogenes were extracted from the dreamBase database (http://rna.sysu.edu.cn/dreamBase/index.php) 48. The miRNA-targeted genes were identified using the miRTarBase (https://mirtarbase.cuhk.edu.cn/~miRTarBase/miRTarBase_2022/php/index.php) 49. The m^6^A-LAIC-seq data of non-tumor cell lines (GM12878 and H1 hESC) were obtained from our previous publication 17, 20. The m^6^A-seq data of non-tumor cell lines (GM12878 21 and H1 hESC 50) were also acquired from previous publications. Expression profiles, clinical files, and anti-PD-L1 therapy information about urothelial cancers were accessed from previous publications 22, 23. Expression profiles of human cancer cell lines (CCLs) were downloaded from the Broad Institute Cancer Cell Line Encyclopedia (CCLE) project (https://portals.broadinstitute.org/ccle/) 51. Drug sensitivity data of CCLs were achieved from the Cancer Therapeutics Response Portal (CTRP v.2.1, https://portals.broadinstitute.org/ctrp.v2.1/) 52-54 and PRISM Repurposing dataset (PRISM 19Q4, https://depmap.org/portal/prism/) 55-58.

### Processing of sequencing data

The RNA-seq, m^6^A-LAIC-seq, and m^6^A-seq raw reads were subject to quality control with Fastqc, removed the adapter with Cutadapt, and dismissed low-quality bases with Trimmomatic according to the standard protocol of sequencing data with default parameters. Then preprocessed reads are conventionally aligned to the hg19 human genome using Hisat2 with default parameters 59. The proper paired and uniquely mapped reads with perfect match except for mismatches at SNPs were used for the downstream analyses. RNA expression was evaluated by Transcripts Per Kilobase of exon model per Million mapped reads (TPM). TPMs of genes were calculated using StringTie2 60. Differential gene expression analyses were performed using the DESeq2 R package. Genes with an adjusted *P*-value < 0.05 (detected by DESeq2 soft) were differentially expressed. Since most of the pseudogenes were not expressed, we first obtained available pseudogenes in all datasets and excluded those with a TPM value less than 1.

### Collation of m^6^A RNA methylation regulators and pseudogenes

We first compiled a whole list of m^6^A RNA methylation regulators from previously published literature, and then restricted the list to the genes with available RNA expression data in the TCGA HNSCC dataset, which ultimately resulted in a total of 24 m^6^A regulators in the current study. These regulators were stratified into 3 categories based on their functions (List S1). Above all, we systematically compared the expression levels of m^6^A regulators between tumor tissues and normal tissues, as well as among different pathological characteristics. Then, the protein-protein interaction (PPI) network based on the STRING database of studied m^6^A regulators was constructed to explore whether there is an interaction between m^6^A regulators. Hereafter, to investigate whether there is co-expression and correlation between m^6^A regulators, a Pearson correlation analysis among m^6^A regulators was executed. Afterward, we compiled a list of pseudogenes from GENCODE, Vega, and Pseudogene.org databases, which were used for the downstream analyses (List S2).

### Screening for m^6^A-associated prognostic pseudogenes by three methods

The m^6^A-associated prognostic pseudogenes were screened using the following three methods, (1) carry out the Pearson correlation analysis of expression between pseudogene in this study with m^6^A regulators, and filter m^6^A-associated pseudogenes under the conditions | correlation coefficient (referred to as *R*) | ≥0.3 and *P* < 0.05; (2) perform the univariate Cox proportional hazard regression to screen prognostic pseudogenes; (3) conduct the multivariate Cox proportional hazard regression based on the second step. Finally, overlapping candidate m^6^A-associated prognostic pseudogenes were identified.

### Construction of the risk score model

Based on the LASSO Cox regression algorithm 61, an L1-penalized regression on the strength of the highest lambda value selected utilizing 1,000 cross-validations ('1-se' lambda) was implemented to further identify the regression coefficients of the candidate m^6^A-associated prognostic pseudogenes. Then we established a survival risk score model through the LASSO coefficients (β) as follows:



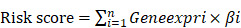



The HNSCC patients were dichotomized into high-risk or low-risk groups based on the median risk score. The receiver operating characteristic (ROC) curve and area under the curve (AUC) was performed to assess the prediction accuracy of the risk score model. Each m^6^A-associated prognostic pseudogene was divided into low or high expression levels, with the cut-off values defined as the median expression value. And Kaplan-Meier plots and Log-rank tests were carried out to estimate and compare the survival rate between subtypes. Then univariate and multivariate Cox regression analyses were utilized to determine the prognostic value of the risk score and various clinical characteristics. Nomograms for evaluating the risk of HNSCC were developed for HNSCC patients based on risk factors. All the analyses mentioned above were conducted using TCGA data as the training set and GEO data as the validation set.

### RNA extraction and real-time quantitative PCR (RT-qPCR) in HNSCC tissues

We applied and collected tumor tissues from 32 HNSCC patients with clinical features, including 16 oral cancer and 16 thyroid cancer tissues, which were from the Tumor Resource Bank of Sun Yat-sen University Cancer Center. Total RNA derived from HNSSC tissues was extracted using the NucleoZol RNA reagent (MACHEREY-NAGEL). And 1 μg of DNA-free RNA was then reverse-transcribed using HiScript III-RT SuperMix for qPCR (+gDNA wiper) (Vazyme, R232). Then qPCR was performed using the ChamQ Universal SYBR qPCR Master Mix (Vazyme, Q711) and carried out in an LC480 Real-Time PCR System (Roche). Ultimately, the fold-change value was calculated using the 2^-∆∆CT^ method. Besides, the clinical features of HNSCC patients and the primers of ten pseudogenes had been appended in List S3 and List S4, respectively.

### Consensus clustering analysis and functional enrichment analysis

To investigate the biological functions of m^6^A-associated prognostic pseudogenes in HNSCC patients, we clustered the patients into different subgroups by the R package “ConsensusClusterPlus” (50 iterations, resample rate of 80%, and Pearson correlation) based on the expression levels of the m^6^A-associated prognostic pseudogenes in TCGA dataset 62. To better understand and interpret the association between m^6^A-associated prognostic pseudogenes and malignancy of HNSCC, GO pathway analysis and GSEA 63 were performed to functionally annotate genes that are differentially expressed in different subgroups by using the R package “clusterProfiler” 64.

### Immune cell infiltration analysis and antitumor immune response analysis

CIBERSORT 45, a bioinformatic deconvolution algorithm to estimate immune cell composition based on related-gene expression profiles, was utilized to calculate tumor-infiltrating cell compositions in HNSCC. The immune cell fractions, expressions of T cell exhausted genes, antigen presentation genes, interferon activity genes, cytolytic genes, integrin genes, and kinase genes were compared in different subtypes and subgroups by Wilcoxon signed-rank test.

### m^6^A-associated pseudogene-miRNA-targeted immune-involved gene regulatory networks

Potential miRNAs binding to pseudogenes were obtained from the dreamBase database 48. Potential miRNA-targeted genes with at least two solid experimental methods (reporter assay and western blot) were extracted using the miRTarBase 49. Pearson analysis was carried out to calculate the expression correlation between m^6^A-associated pseudogenes and miRNA-targeted genes. Then these targeted genes conforming to | *R* | **≥** 0.3 and *P* < 0.05 were identified and applied to build pseudogene-miRNA-targeted immune-involved gene regulatory networks.

### Cell culture

ARO, Tca8113, and HeLa cells were cultured in Roswell Park Memorial Institute (RPMI) Medium 1640 (Corning), supplemented with 10% fetal bovine serum (FBS) (Gibco) at 37 °C with 5% CO_2_. GSCs-U251 and MDA-MB-231 cells were cultured in Dulbecco's Modification of Eagle's Medium (DMEM) (Corning), supplemented with 10% fetal bovine serum (FBS) (Gibco) at 37 °C with 5% CO_2_. All cells were tested to ensure that they are free from Mycoplasma infection.

### siRNA and miRNA inhibitor transfection

Cells were seeded in TC-untreated plates and transfected with siNC and specific siRNA or inhibitor NC and specific miRNA inhibitor using RNAiMAX (Invitrogen) according to the manufacturer's instructions. RNA samples and protein samples were harvested at 72 h after transfection for qRT-PCR or western blotting. The siRNA pools targeting PDIA3P1 and RRN3P3, as well as control siRNA (NC), were synthesized by RIBOBIO (Guangzhou, China). The siRNA duplexes used in the current study are listed in List S5.

### RNA extraction and RT-qPCR in HNSCC cells

Total RNA from HNSCC cells was extracted using the NucleoZol RNA reagent (MACHEREY-NAGEL). And the specific steps of RT-qPCR are the same as in Part “RNA extraction and real-time quantitative PCR (RT-qPCR) in HNSCC tissues”**.** In addition, the primers of pseudogenes and their targeted immune-involved genes had been appended in List S4.

### Western blotting

Proteins were extracted from cells after incubating with RIPA buffer (Cell Signaling Technology, Cat. 9806) on ice for 10 min, and then the insoluble fraction was removed by centrifugation. 20 μg of extracted protein was separated on 15% SDS-PAGE and transferred to the PVDF membrane. The membranes were blocked in 5% BSA in Tris-Buffered Saline with 0.01% Tween 20 (TBS-T) at room temperature for 1 h and incubated overnight with primary antibodies diluted in 1% BSA/TBS-T at 4 °C, followed by incubating with goat anti-rabbit HRP conjugated secondary antibody diluted in TBS-T for 1 h at room temperature and visualized using Clarity™ Western ECL Substrate (Bio-Rad). The following antibodies were used for immunoblotting: AKT1 (1:1000, Affinity Biosciences, AF0836-50), and EZH2 (1:1000, Affinity Biosciences, AF5150-50).

### RNA stability assay

The ultimate concentration of 5 μg/mL Actinomycin D (Sigma, A9415) was added to control and *PDIA3P1*/*RRN3P3* knockdown cells to assess RNA stability. The cells were collected after incubation for indicated time points, and RNA samples were extracted for reverse transcription and qPCR. And 18S was utilized as the reference gene and the fold-change value was calculated using the 2^-ΔΔCT^ method.

### m^6^A analyses about m^6^A-associated pseudogene

We recalculated the m^6^A levels of all compiled annotated pseudogenes based on the reprocessed m^6^A-LAIC-seq profiles of non-tumor cells (GM12878 and H1 cells) according to the method described in our previously published paper 17, 20 and identified the m^6^A peaks based on the reprocessed m^6^A-seq profiles of non-tumor cells (GM12878 21 and H1 50). In addition, the single-nucleotide m^6^A sites were determined by combining the m^6^A sites predicted by sequence-based m^6^A site predictors SRAMP 65 and Whistle 66 within m^6^A peak regions.

### Absolute quantification of m^6^A modification on pseudogene mRNA

We adopted the Epi-SELECT^TM^ m^6^A fraction quantification kit (Epibiotek) to detect absolute m^6^A levels on pseudogenes in ARO and Tca8113 cells. The experimental schematic and protocol of SELECT (single base elongation- and ligation-based qPCR amplification method) were performed as previously described 67. In brief, 9.8 μl total RNA was mixed with 1.6 μl Up Primer (1 μM), 1.6 μl Down Primer (μM), and 2 μl dNTP in a 2 μl 10× Reaction Buffer. The RNA and primers were annealed by incubating the mixture at a temperature gradient: 90 °C for 1 min, 80 °C for 1 min, 70 °C for 1 min, 60 °C for 1 min, 50 °C for 1 min, and then 40 °C for 6 min. Subsequently, 3 μl of a mixture containing 0.3 μl SELECT^TM^ DNA polymerase, 0.47 μl SELECT^TM^ ligase, and 2.23 μl ATP was added to the former mixture to a final volume of 20 μl. The final reaction mixture was incubated at 40 °C for 20 min and denatured at 80 °C for 20 min. The qPCR reaction was performed using ChamQ Universal SYBR qPCR Master Mix (Vazyme, Q711) with the Roche Lightcycler 480 Instrument II system. According to the above methods, the experiments of the control and METTL3 knockout group were carried out. SELECT primer sequences and shMETTL3 sequences are listed in List S6.

### Comprehensive analysis of ICI therapy benefit

To explore the prognostic value of m^6^A-associated pseudogene in HNSCC patients after immunotherapy, we compared the immune dysfunction and exclusion (TIDE) score, MSI, T cell dysfunction, TAM_M2, T cell exclusion score, MASC, and CAF, which were calculated online (http://tide.dfci.harvard.edu/) among different subtypes 68. Besides, the tumor inflammation signature (TIS) score 69 was calculated as an average value of log2-scale normalized expression of the 18 signature genes (List S7) and compared in different subtypes. Moreover, four methods (ABSOLUTE, IHC, CPE, and ESTIMATE) 70-72 were performed to estimate the purity of the tumor, which can better understand ICI therapy's benefit. To validate the prognostic value of m^6^A-associated pseudogene in patients after immunotherapy, we performed survival analyses in two urothelial cancer (UC) cohorts treated with PD-L1 blockade 22, 23. Moreover, we performed time-dependent ROC curve analyses to obtain AUC which is used to evaluate the prognostic value of m^6^A-associated pseudogene with the R package of timeROC.

### Predictive analysis of potential therapeutic agents for patients with high-risk scores HNSCC

Drug sensitivity profiles of cancer cell lines (CCLs) were downloaded from the Cancer Therapeutics Response Portal (CTRP v.2.0, https://portals.broadinstitute.org/ctrp/) and Profiling Relative Inhibition Simultaneously in Mixtures dataset (PRISM 19Q4, https://depmap.org/portal/prism/). At first, compounds with more than 20% of missing data were excluded. After imputation, the K-nearest neighbor (k-NN) imputation was used to impute the missing AUC values. Based on CTRP and PRISM-derived drug response profiles respectively, the two different approaches were determined to identify candidate agents with higher drug sensitivity in high-risk score patients (Figure S12B). Then the differences in the mRNA and protein expression levels of candidates' drug targets between tumor and normal tissue were calculated, and a higher differential (fold change) value indicated a greater potential and better efficacy of the candidate agent. The CMap analysis was utilized to select agents in which gene expression patterns were contrary to the HNSCC-specific expression patterns 73. The drug data query was performed in DrugBank (https://go.drugbank.com/) and the literature search was conducted in PubMed (https://www.ncbi.nlm.nih.gov/pubmed/), which was used to seek the experimental validation and clinical evidence of candidate agents in treating HNSCC. The code for the predictive analysis of this section was available at https://github.com/tlqsysu/Predictive-analysis-of-potential-therapeutic-agents-for-patients-with-high-risk-scores-HNSCC.

### MTT assay

Cell viability was estimated by using the 3-(4,5-dimethylthiazol-2-yl)-2,5-diphenyltetrazolium bromide (MTT; Sigma, St Louis, USA) colorimetric assay. In short, cells were seeded in 96-well plates and incubated for 24 h, and cells were treated with the drug at different concentrations. After the treatments, cells were washed with phosphate-buffered saline (PBS) and then incubated in MTT solution (Sigma) for 4 h. After dimethyl sulfoxide (DMSO) was added to each well, the absorbance value was measured at 490 nm to decide the cell viability with a microplate reader (BioTek, Winooski, USA). The cell viability was plotted in a graph and the IC_50_ was calculated accordingly to determine the optimum dosage of the drugs for further studies.

### Statistical Analysis

One-way ANOVA and t-test were conducted to compare the expression levels of m^6^A-associated prognostic pseudogenes in different subtypes and subgroups differentiated by different clinical characteristics. A Chi-square test was utilized to estimate the differences in clinicopathological characteristics in different subtypes and subgroups identified by consensus clustering of pseudogenes. All statistical analyses were carried out using R4.0 software (http://www.r-project.org/) and Bioconductor (http://bioconductor.org/). A two-sided *P* value < 0.05 was considered statistically significant in all analyses.

## Supplementary Material

Supplementary figures, list and table legends.

Supplementary lists.

Supplementary tables.

## Abbreviations

TCGA: The Cancer Genome Atlas
GEO: Gene Expression Omnibus
LASSO: the least absolute shrinkage and selection operator
ROC: the receiver operating characteristic curve
AUC: area under the curve
PDIA3P1: protein disulfide isomerase family A member 3 pseudogene 1
LDHAP4: lactate dehydrogenase A pseudogene 4
LDHAP7: lactate dehydrogenase A pseudogene 7
EEF1A1P6: eukaryotic translation elongation factor 1 alpha 1 pseudogene 6
EEF1A1P11: eukaryotic translation elongation factor 1 alpha 1 pseudogene 11
SDHAP1: succinate dehydrogenase complex flavoprotein subunit A pseudogene 1
SDHAP3: succinate dehydrogenase complex flavoprotein subunit A pseudogene 3
DDX12P: DEAD/H-Box Helicase 12 pseudogene
CLUHP3: clustered mitochondria homolog pseudogene 3
RRN3P3: homolog, RNA polymerase I transcription factor pseudogene 3
P1: patient subgroup 1
P2: patient subgroup 2
ceRNA: competitive endogenous RNA
PD-1: programmed cell death 1
PD-L1: programmed cell death 1 ligand 1
PD-L2: programmed cell death 1 ligand 2
LAG3: lymphocyte-activation gene 3
TIGIT: T cell immunoreceptor with Ig and ITIM domains
CTLA4: cytotoxic T-lymphocyte-associated protein 4
HLA: major histocompatibility complex, class I
TAP1: transporter 1, ATP binding cassette subfamily B member
B2M: beta-2-microglobulin
CCL5: C-C motif chemokine ligand 5
CXCL9: C-X-C motif chemokine ligand 9
CD24: CD24 molecule
CD27: CD27 molecule
STAT1: signal transducer and activator of transcription 1
IRF3: interferon regulatory factor 3
GZMA: Granzyme A
PRF1: Perforin 1
CYTH: cytohesin
ITGA: integrin subunit alpha
ITGB: integrin subunit beta
AKT1: AKT serine/threonine kinase 1
FOXM1: forkhead box M1
E2F2: E2F transcription factor 2
MECP2: methyl-CpG binding protein 2
MAGEA: MAGE family member A
HOXA: homeobox A
EZH2: Enhancer Of Zeste 2 Polycomb Repressive Complex 2 Subunit
GO: Gene Ontology
GSEA: Gene Set Enrichment Analysis
TIDE: tumor immune dysfunction and exclusion
MSI: microsatellite instability
TAM: tumor-associated macrophages
MDSC: myeloid-derived suppressor cell
CAF: cancer-associated fibroblasts
TIS: tumor inflammation signature
IHC: immunohistochemistry
CPE: consensus measurement of purity estimations
ESTIMATE: Estimation of Stromal and Immune cells in Malignant Tumours using Expression data
CCLs: cancer cell lines
CTRP: Cancer Therapeutics Response Portal
PRISM: Profiling Relative Inhibition Simultaneously in Mixtures
