# Supplementary material for: N6-methyladenosine-associated prognostic pseudogenes contribute to predicting immunotherapy benefits and therapeutic agents in head and neck squamous cell carcinoma
Source: Theranostics. 2022 Oct 17;12(17):7267–88. doi: 10.7150/thno.76689 (PMC9691354; doi:10.7150/thno.76689)
Supplement: Supplementary file 1 — Supplementary figures, list and table legends. [file thnov12p7267s1.pdf]

Supplementary Material

Supplementary Figures

Figure S1. The workflow in this study. **Figure S2.** Expression of 24 m^{6}A regulators between tumor tissues and corresponding normal tissues (n = 44) in TCGA dataset. **Figure S3.** Cox proportional hazard regression of GEO dataset and LASSO regression of TCGA dataset about 10 m^{6}A-associated prognostic pseudogenes. **Figure S4.** Boxplot comparing the expression levels of the 10 m^{6}A-associated pseudogenes in HNSCC patients with different HPV status and histologic grades in the TCGA dataset. **Figure S5.** Boxplot comparing the expression levels of the 10 m^{6}A-associated pseudogenes in HNSCC patients with different pathology TNM stages in the TCGA dataset. **Figure S6.** Kaplan-Meier curves show the association between the expression levels of m^{6}A regulators and m^{6}A-associated pseudogenes and overall survival in patients with HNSCC from the GEO dataset. **Figure S7.** Expressions of 10 m^{6}A-associated pseudogenes are significantly associated with survival outcomes of HNSCC patients from the TCGA dataset. **Figure S8.** Comparisons of the expressions of integrin ITGA family genes, integrin ITGB family genes, and kinase genes between low-risk and high-risk subtypes. **Figure S9.** The expression pattern of m^{6}A-associated prognostic pseudogenes was significantly correlated with an antitumor immune response between the P1 and P2 subgroups. **Figure S10.** Functional enrichment analysis of differentially expressed genes between P1 and P2 subgroups. **Figure S11.** m^{6}A-associated pseudogenes can regulate targeted immune-involved genes via miRNAs. **Figure S12.** Identification of candidate agents

with higher drug sensitivity in high-risk score patients with HNSCC.

Supplementary Lists

List S1. 24 m^6A regulators included in the current study. **List S2.** Pseudogenes included in the current study. **List S3.** The clinical features of HNSCC patients in our hospital dataset used in the current study. **List S4.** RT-qPCR primer sequences used in the current study (sequences are from 5’ to 3’). **List S5.** siRNA sequences used in the current study. **List S6.** SELECT primer sequences and shMETTL3 sequences used in the current study. **List S7.** Tumor Inflammation signatures (TIS) included in the current study.

Supplementary Tables

Table S1. m^6A-associated pseudogenes correlated with their m^6A regulators at $|R| \geqslant 0.3$ and $P < 0.05$. **Table S2.** Identification of prognostic pseudogenes by the univariate Cox proportional hazard regression. **Table S3.** The coefficients of the 10 m^6A-associated prognostic pseudogenes by LASSO. **Table S4.** Clinicopathological features stratified by high-risk and low-risk subtypes. **Table S5.** Clinicopathological features stratified by P1 and P2 subgroups. **Table S6.** Potential miRNAs binding to the 10 m^6A-associated prognostic pseudogenes identified by dreamBase. **Table S7.** miRNA targeted genes correlated with their pseudogenes at $|R| \geqslant 0.3$ and $P < 0.05$. **Table S8.** Tumor Inflammation Signature (TIS) Score. **Table S9.** Drug sensitivity estimated by AUC from CTRP. **Table S10.** Drug sensitivity estimated by AUC from

PRISM. **Table S11.** The fold-change differences in the mRNA expression level of candidates' drug targets between tumor and normal tissue. **Table S12.** The CMap Score of compounds.

Supplementary file 1: Figure S1 to S12 for

N6-methyladenosine-associated prognostic pseudogenes contribute to predicting immunotherapy benefits and therapeutic agents in head and neck squamous cell carcinoma

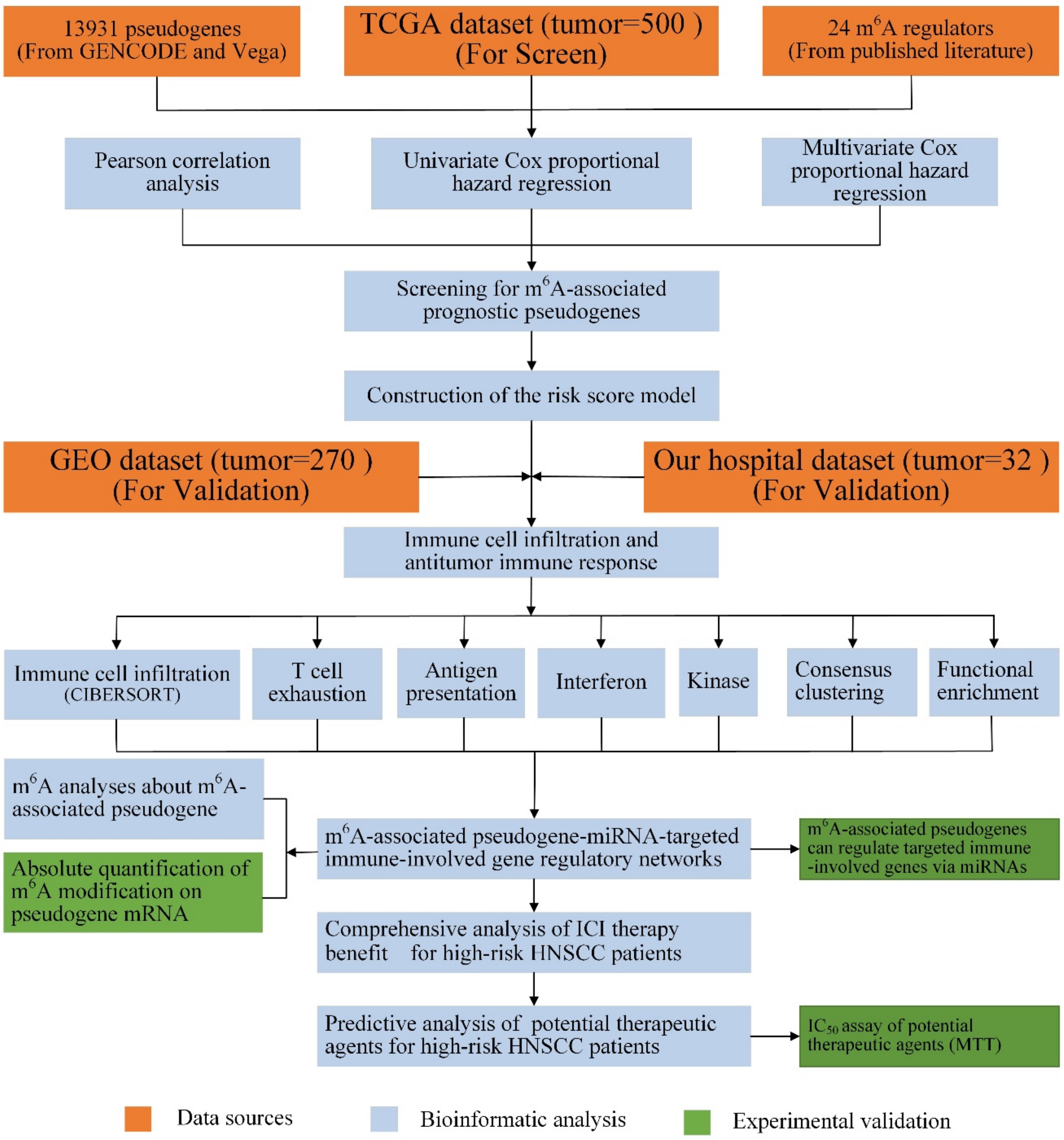

Figure S1. The workflow in this study.

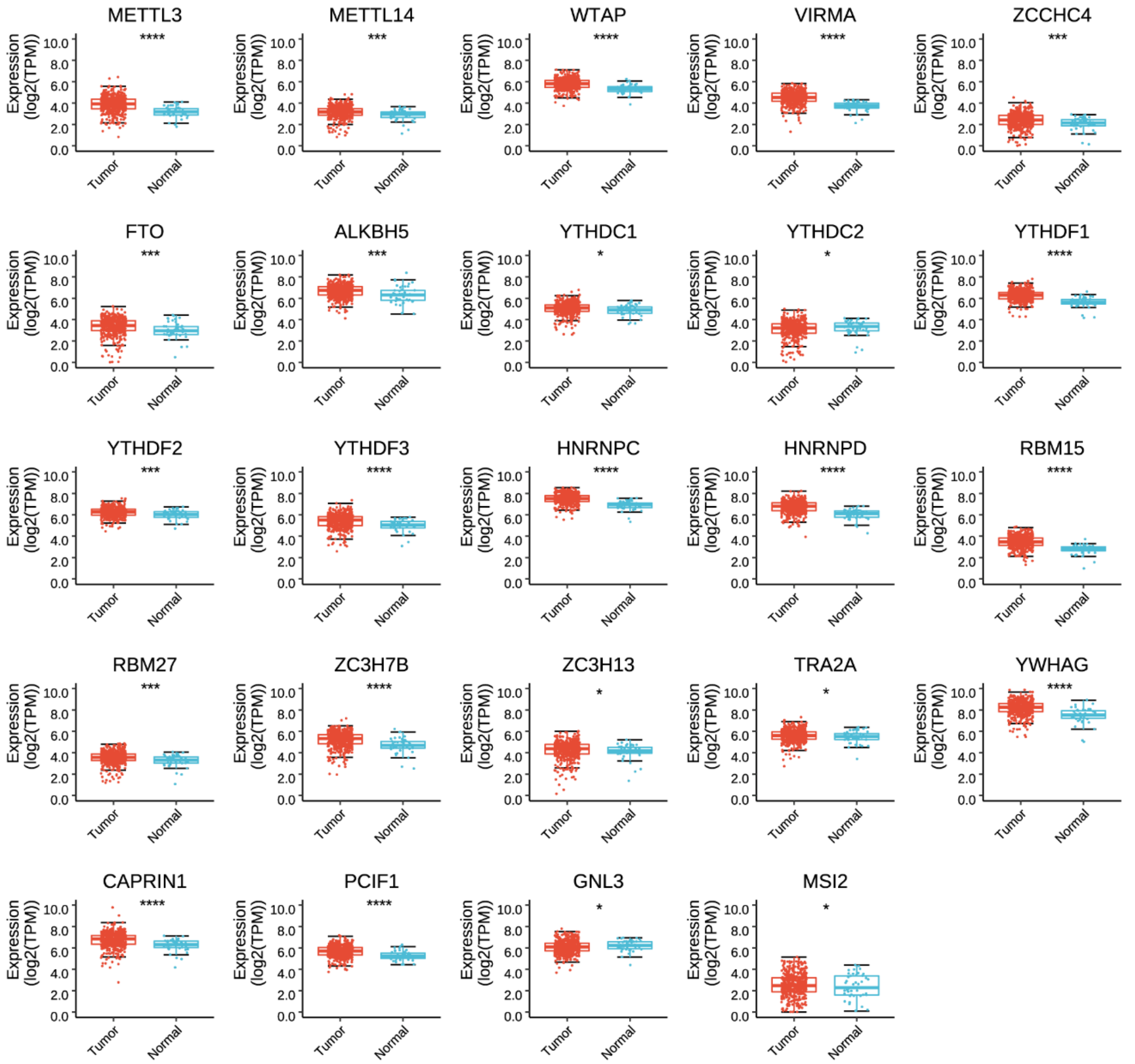

Figure S2. Expression of 24 m^{6}A regulators between tumor tissues and corresponding normal tissues (n = 44) in TCGA dataset. Boxplot comparing the expression distribution of *METTL3*, *METTL14*, *WTAP*, *VIRMA*, *ZCCHC4*, *FTO*, *ALKBH5*, *YTHDC1*, *YTHDC2*, *YTHDF1*, *YTHDF2*, *YTHDF3*, *HNRNPC*, *HNRNPD*, *RBM15*, *RBM27*, *ZC3H7B*, *ZC3H13*, *TRA2A*, *YWHAG*, *CAPRIN1*, *PCIF1*, *GNL3* and *MSI2* between tumor and corresponding normal tissues. The expression value between tumor and normal tissues were compared through the Wilcoxon test. ns denotes no significance, * denotes $P < 0.05$, ** denotes $P < 0.01$, *** denotes $P < 0.001$ and ****

denotes $P < 0.0001$.

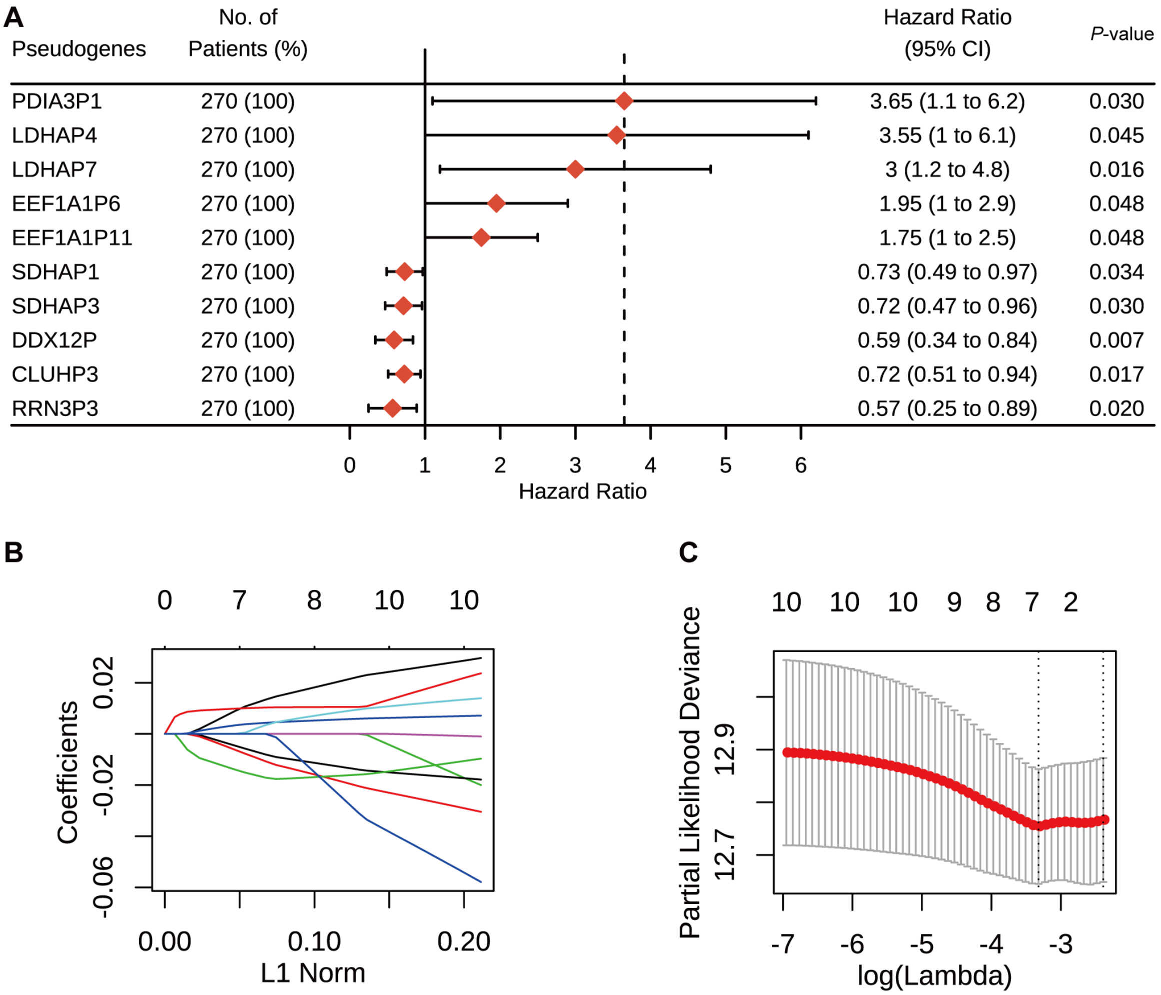

Figure S3. Cox proportional hazard regression of GEO dataset and LASSO regression of TCGA dataset about 10 m^{6}A-associated prognostic pseudogenes. (A) Forest plot showing the hazard ratios (HR), and 95% confidence intervals (CI) calculated by univariate Cox proportional hazard regression of the 10 m^{6}A-associated prognostic pseudogenes in the GEO dataset. (B) The plot of LASSO coefficient profiles of 10 m^{6}A-associated prognostic pseudogenes in the TCGA dataset. (C) The plot of ten-time cross-validation for tuning parameter selection in the LASSO model of 10 m^{6}A-associated prognostic pseudogenes in the TCGA dataset.

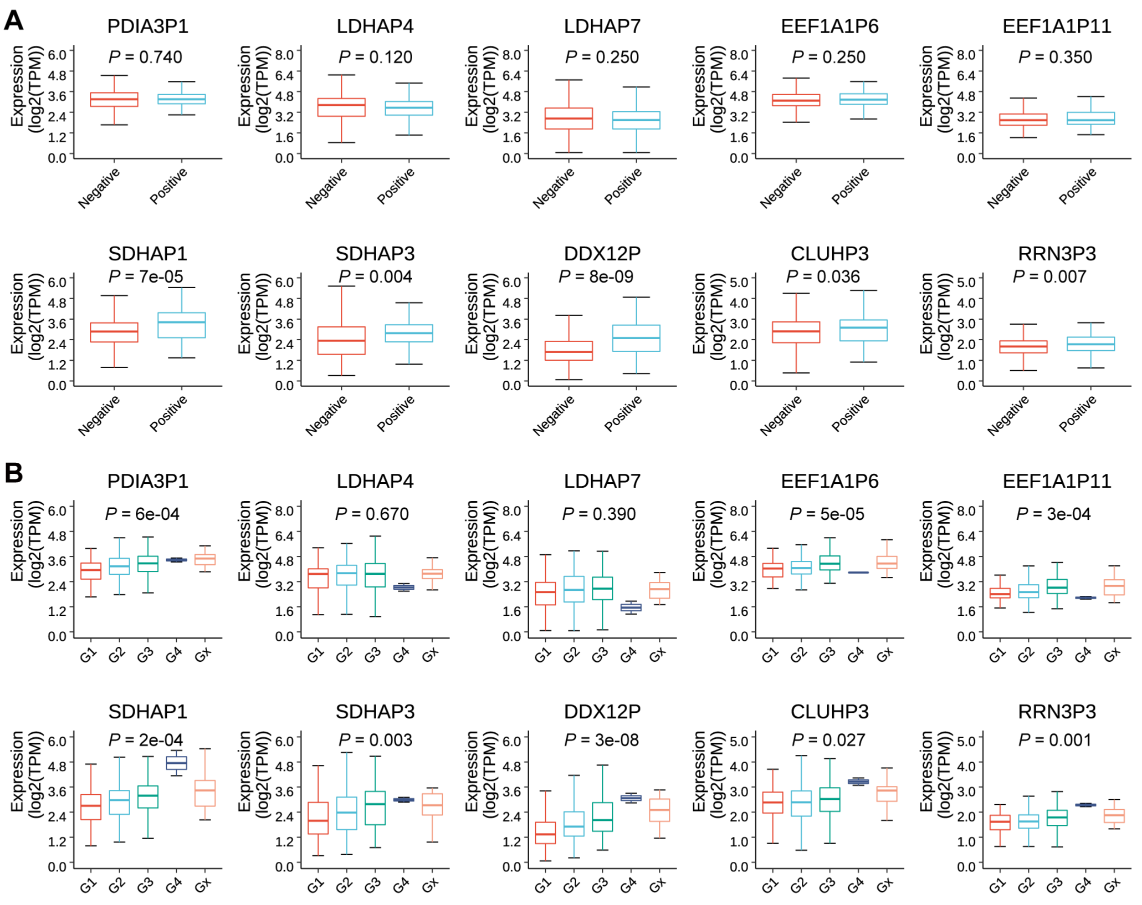

Figure S4. Boxplot comparing the expression levels of the 10 m^{6}A-associated pseudogenes in HNSCC patients with different HPV status and histologic grades in the TCGA dataset. (A) Boxplot comparing the expression levels of *PDIA3P1*, *LDHAP4*, *LDHAP7*, *EEF1A1P6*, *EEF1A1P11*, *SDHAP1*, *SDHAP3*, *DDX12P*, *CLUHP3*, and *RRN3P3* in HNSCC patients with between HPV positive and HPV negative. (B) Boxplot comparing the expression levels of *PDIA3P1*, *LDHAP4*, *LDHAP7*, *EEF1A1P6*, *EEF1A1P11*, *SDHAP1*, *SDHAP3*, *DDX12P*, *CLUHP3*, and *RRN3P3* in HNSCC patients across the different histologic grades.

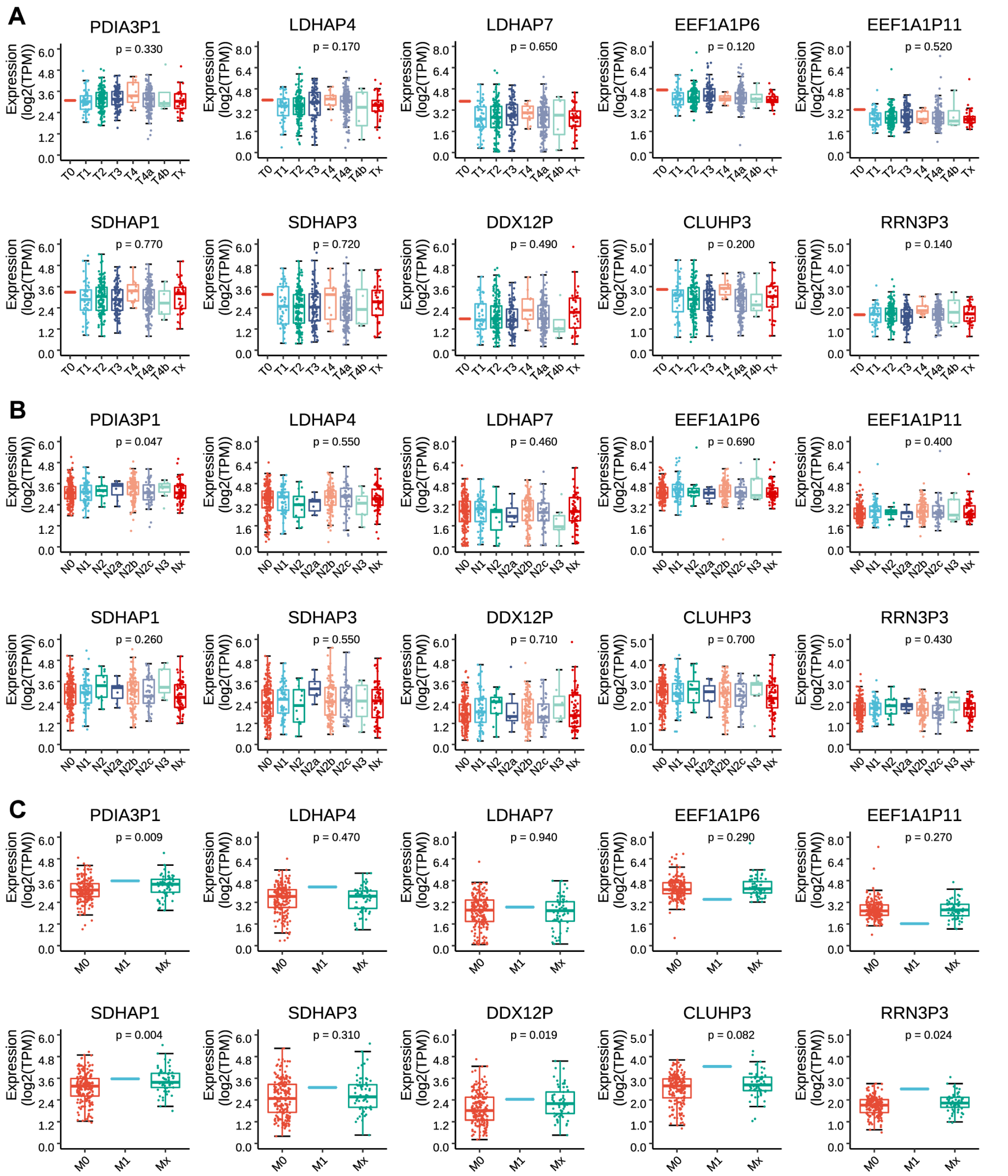

Figure S5. Boxplot comparing the expression levels of the 10 m^{6}A-associated pseudogenes in HNSCC patients with different pathology TNM stages in the TCGA dataset. (A) Boxplot comparing the expression levels of *PDIA3P1*, *LDHAP4*, *LDHAP7*, *EEF1A1P6*, *EEF1A1P11*, *SDHAP1*, *SDHAP3*, *DDX12P*, *CLUHP3*, and *RRN3P3* in HNSCC patients with different pathology T stage. (B) Boxplot comparing the expression

levels of *PDIA3P1*, *LDHAP4*, *LDHAP7*, *EEF1A1P6*, *EEF1A1P11*, *SDHAP1*, *SDHAP3*, *DDX12P*, *CLUHP3,* and *RRN3P3* in HNSCC patients with different pathology N stage. (C) Boxplot comparing the expression levels of *PDIA3P1*, *LDHAP4*, *LDHAP7*, *EEF1A1P6*, *EEF1A1P11*, *SDHAP1*, *SDHAP3*, *DDX12P*, *CLUHP3*, and *RRN3P3* in HNSCC patients with different pathology M stages.

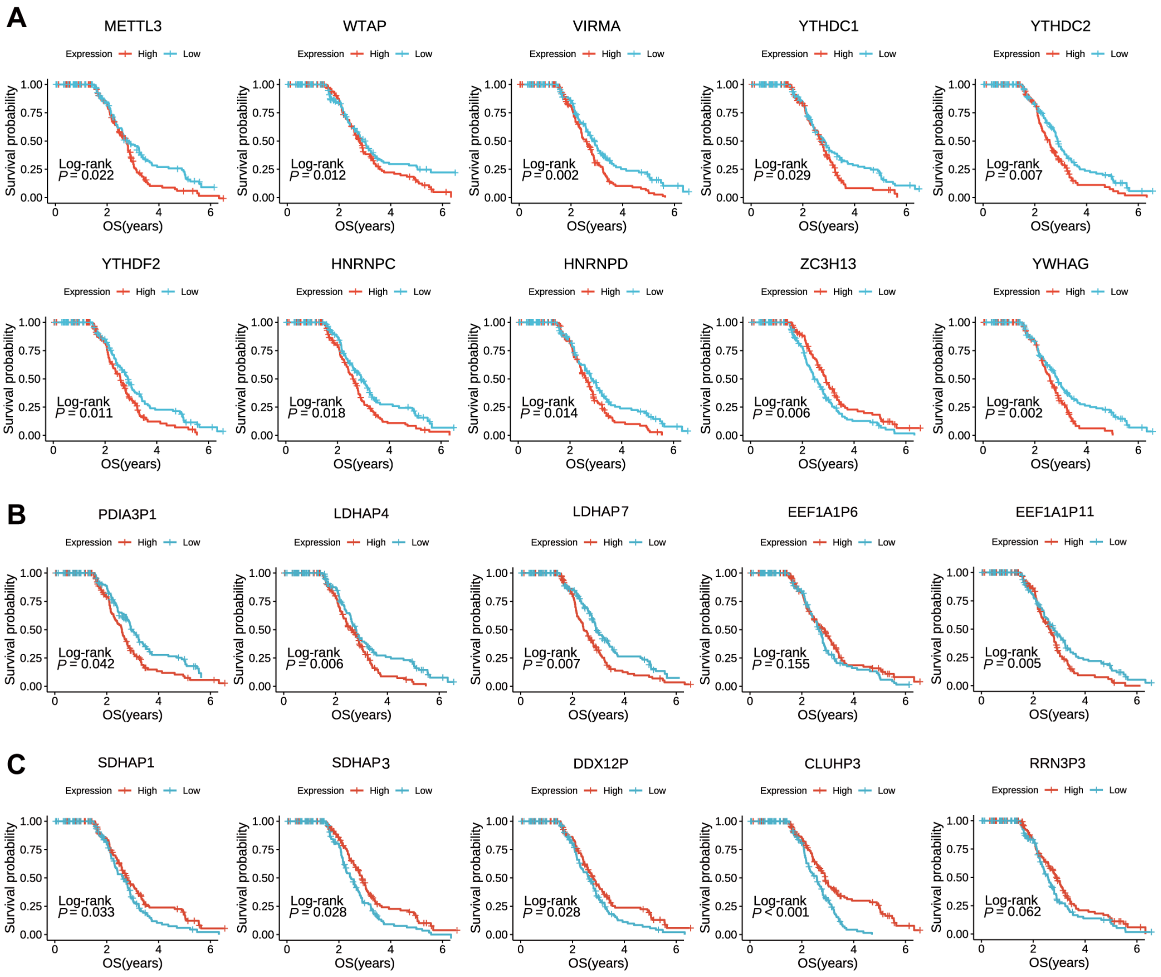

Figure S6. Kaplan-Meier curves show the association between the expression levels of m^{6}A regulators and m^{6}A-associated pseudogenes and overall survival in patients with HNSCC from the GEO dataset. (A) Kaplan-Meier curves of association between the expression levels of m^{6}A regulators and overall survival in patients with HNSCC from the GEO dataset. (B) Kaplan-Meier curves of association between the expression levels of oncogene pseudogenes and overall survival in patients with HNSCC from the GEO dataset. (C) Kaplan-Meier curves of association between the expression levels of tumor-suppressor pseudogenes and overall survival in patients with HNSCC from the GEO dataset.

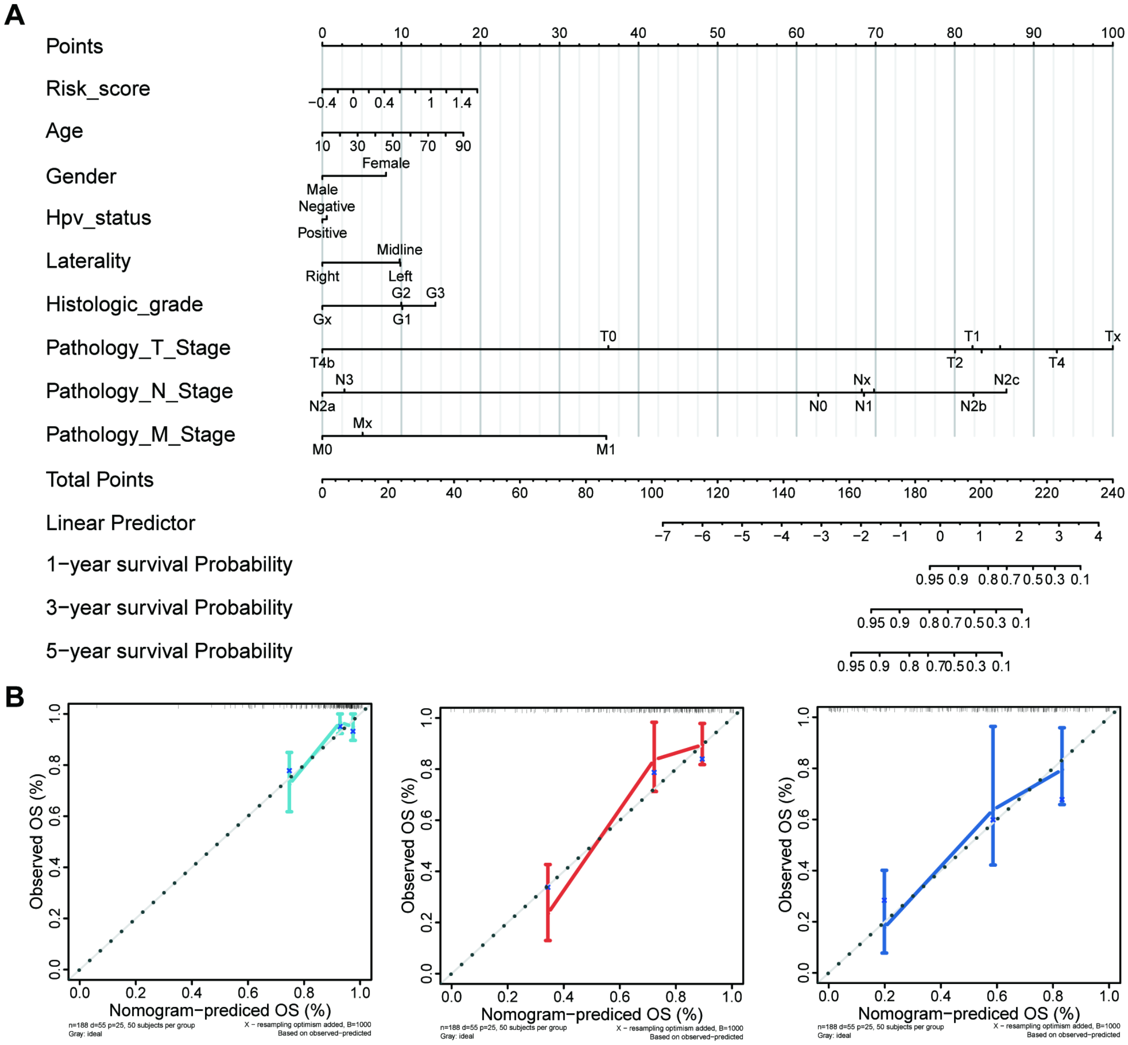

Figure S7. Expressions of 10 m^{6}A-associated pseudogenes are significantly associated with survival outcomes of HNSCC patients from the TCGA dataset. (A) Nomogram for predicting the 1-year, 3-year, and 5-year prognosis of HNSCC patients from the TCGA dataset. The nomogram was applied by summing the points identified on the points scale for each variable. According to the total points on the bottom scales, the nomogram provides the probability of the 1-year, 3-year, and 5-year prognosis for an individual patient. (B) Calibration curves of the nomogram for predicting the 1-year, 3-year, and 5-year prognosis of HNSCC patients from the TCGA dataset, respectively. The X-axis represents the nomogram-predicted probability of progression, and the Y-axis

represents the actual probability estimated with the Kaplan-Meier method. The light blue, orange, and dark blue line represents the ideal correlation between the nomogram-predicted and actual probability of 1-year, 3-year, and 5-year, respectively.

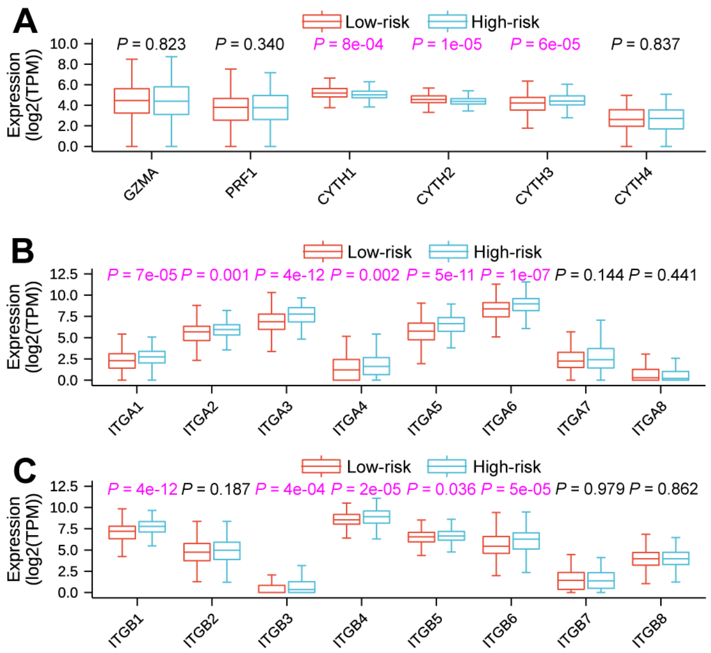

Figure S8. Comparisons of the expressions of integrin ITGA family genes, integrin ITGB family genes, and kinase genes between low-risk and high-risk subtypes. (A) Boxplot contrasting the expressions of *GZMA*, *PRF1*, *CYTH1*, *CYTH2*, *CYTH3*, and *CYTH4* between low-risk and high-risk subtypes. (B) Boxplot comparing the expressions of ITGA family genes between low-risk and high-risk subtypes. (C) Boxplot comparing the expressions of ITGB family genes between low-risk and high-risk subtypes. The *P*-value of comparisons between the two subtypes was calculated through the Wilcoxon test. Purple represents *P*-value < 0.05.

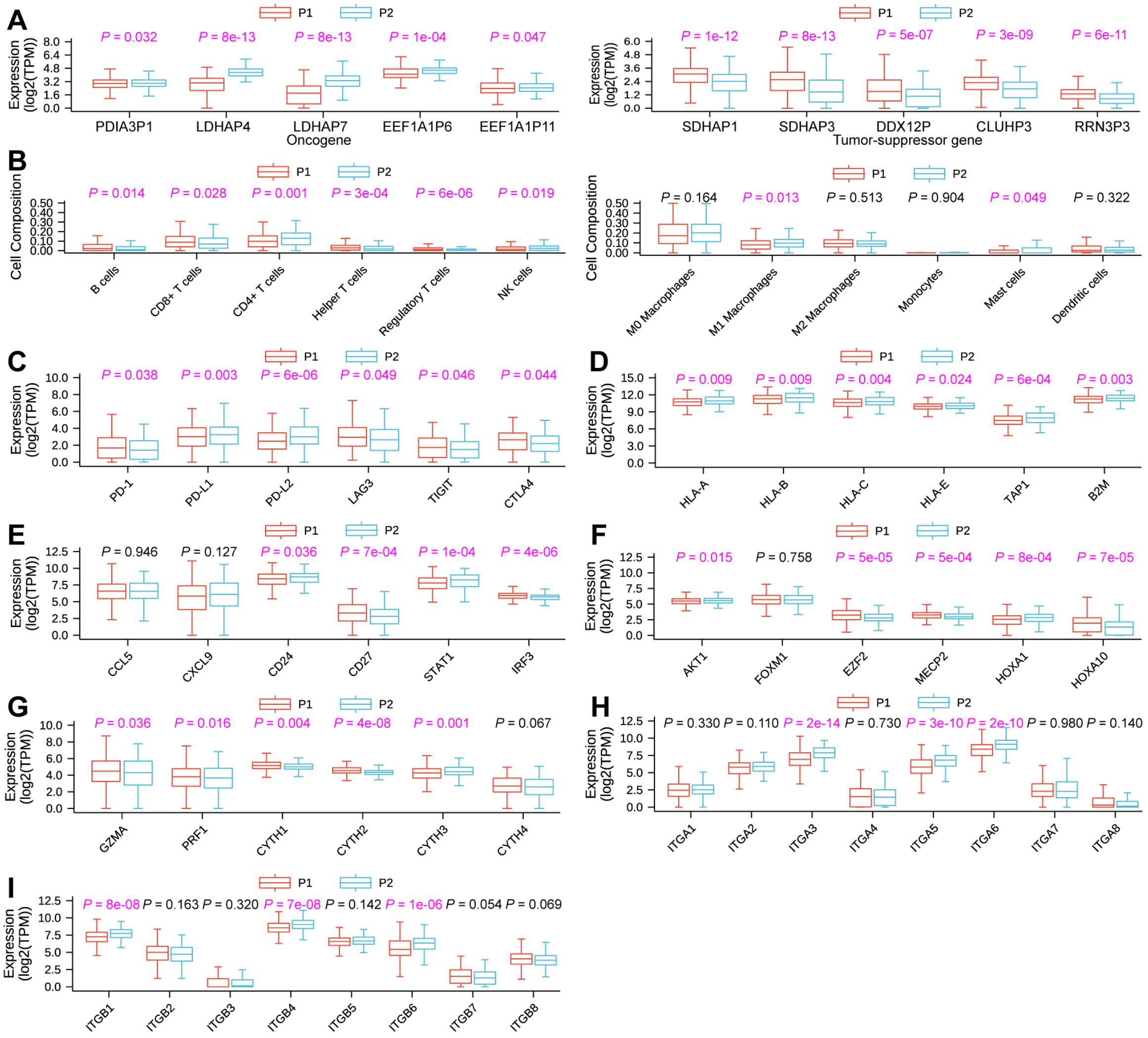

Figure S9. The expression pattern of m^{6}A-associated prognostic pseudogenes was significantly correlated with an antitumor immune response between the P1 and P2 subgroups. (A) Boxplot revealing comparisons of expression levels of oncogenes (*PDIA3P1*, *LDHAP4*, *LDHAP7*, *EEF1A1P6*, *EEF1A1P11*) and tumor-suppressor genes (*SDHAP1*, *SDHAP3*, *DDX12P*, *CLUHP3*, *RRN3P3*) between P1 and P2 subgroups. (B) Boxplot showing comparisons of cell composition fraction of B cells, CD8+ T cells, CD4+ T cells, helper T cells, regulatory T cells, activated natural killer (NK) cells, M0 macrophages, M1 macrophages, M2 macrophages, monocytes, mast cells, and activated dendritic cells between P1 and P2 subgroups. (C) Boxplot displaying comparisons of

expressions of *PD-1*, *PD-L1*, *PD-L2*, *LAG3*, *TIGIT*, and *CTLA4* between P1 and P2 subgroups. (D) Boxplot manifesting comparisons of expressions of *HLA-A*, *HLA-B*, *HLA-C*, *HLA-E*, *TAP1*, and *B2M* between P1 and P2 subgroups. (E) Boxplot comparing the expressions of *CCL5*, *CXCL9*, *CD24*, *CD27*, *STAT1*, and *IRF3* between P1 and P2 subgroups. (F) Boxplot comparing the expressions of kinase genes (*AKT1*, *FOXM1*, *E2F2*, *MECP2*, *HOXA1*, and *HOXA10*) between P1 and P2 subgroups. (G) Boxplot contrasting the expressions of *GZMA*, *PRF1*, *CYTH1*, *CYTH2*, *CYTH3*, and *CYTH4* between P1 and P2 subgroups. (H) Boxplot comparing the expressions of ITGA family genes between P1 and P2 subgroups. (I) Boxplot comparing the expressions of ITGB family genes between P1 and P2 subgroups. The *P*-value of comparisons between the two subgroups was calculated through the Wilcoxon test. Purple represents *P*-value < 0.05.

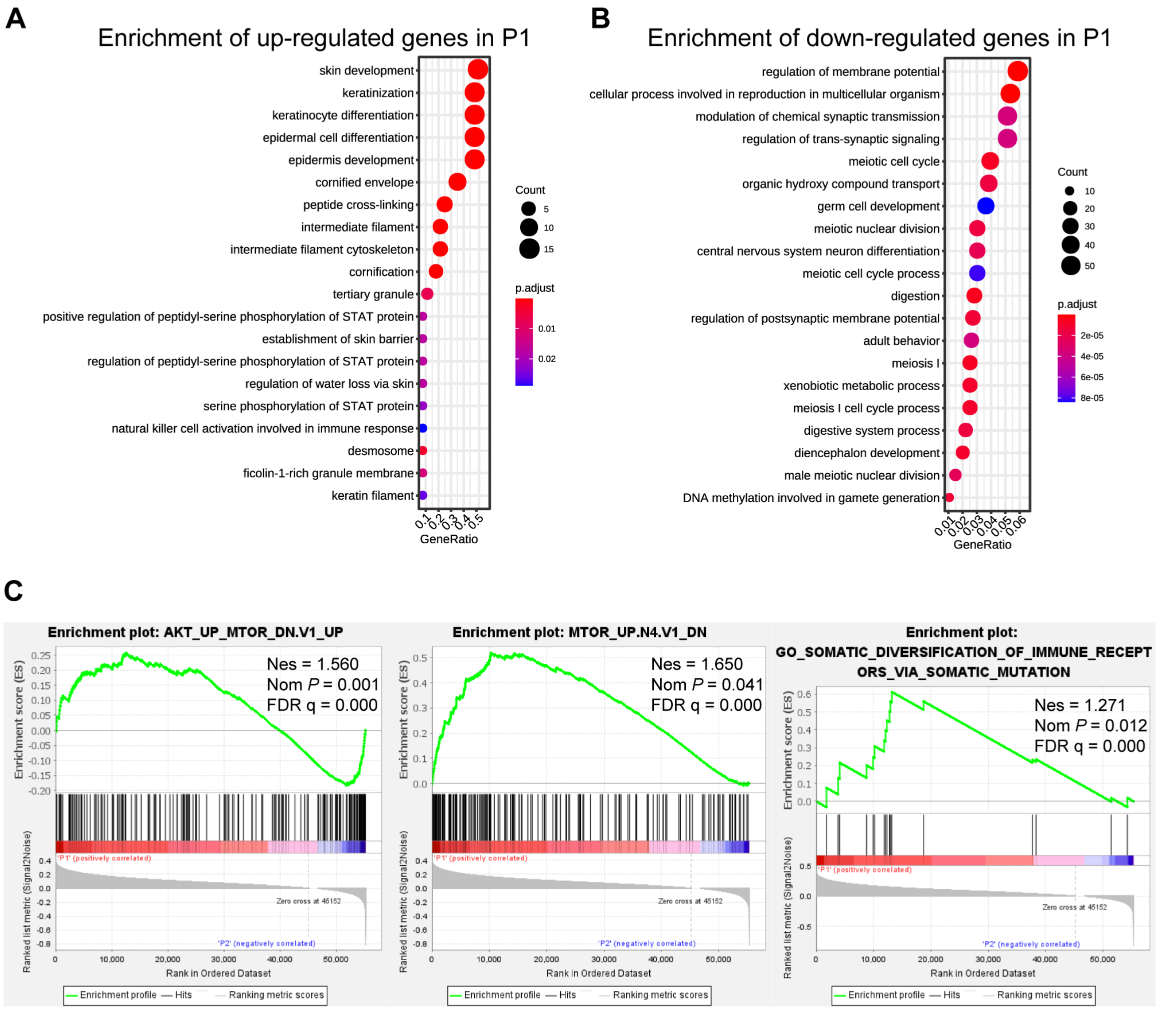

Figure S10. Functional enrichment analysis of differentially expressed genes between P1 and P2 subgroups. (A) Functional enrichment analysis of up-regulated genes in the P1 subgroup compared with P2 by using GO in terms of biological process signaling pathway. (B) Functional enrichment analysis of down-regulated genes in the P1 subgroup compared with P2 by using GO in terms of biological process signaling pathway. The GO and pathway terms are displayed on the x-axis and are significantly enriched at −log10 (*P*-value). (C) Gene set enrichment analysis (GSEA) revealed that up-regulated genes in the P1 subgroup were enriched for hallmarks of malignant tumors.

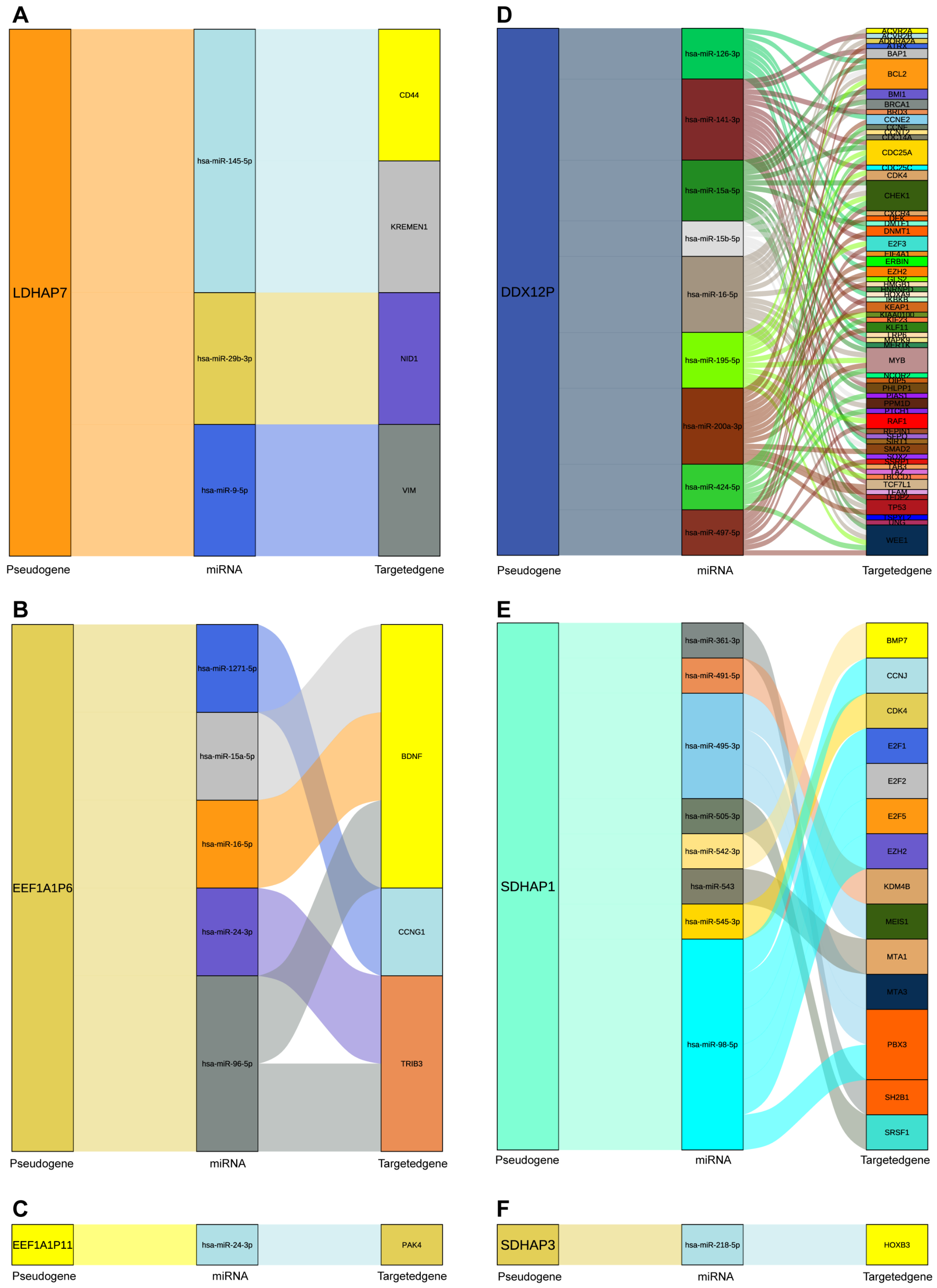

Figure S11. m^{6}A-associated pseudogenes can regulate targeted immune-related

genes via miRNAs. (A-F) Sankey plots showing pseudogenes together with binding miRNAs and target genes with $| r | \geq 0.3$ and $P < 0.05$ were used to construct the pseudogene-miRNA-target gene regulatory networks by subtypes of oncogene pseudogene *LDHAP7* (A), *EEF1A1P6* (B), *EEF1A1P11* (C), and tumor-suppressor pseudogene *DDX12P* (D), *SDHAP1* (E), *SDHAP3* (F). The column on the left represented pseudogenes, which are located at the cores of the networks. The column in the middle and the column on the right stand for binding miRNAs and target genes, respectively.

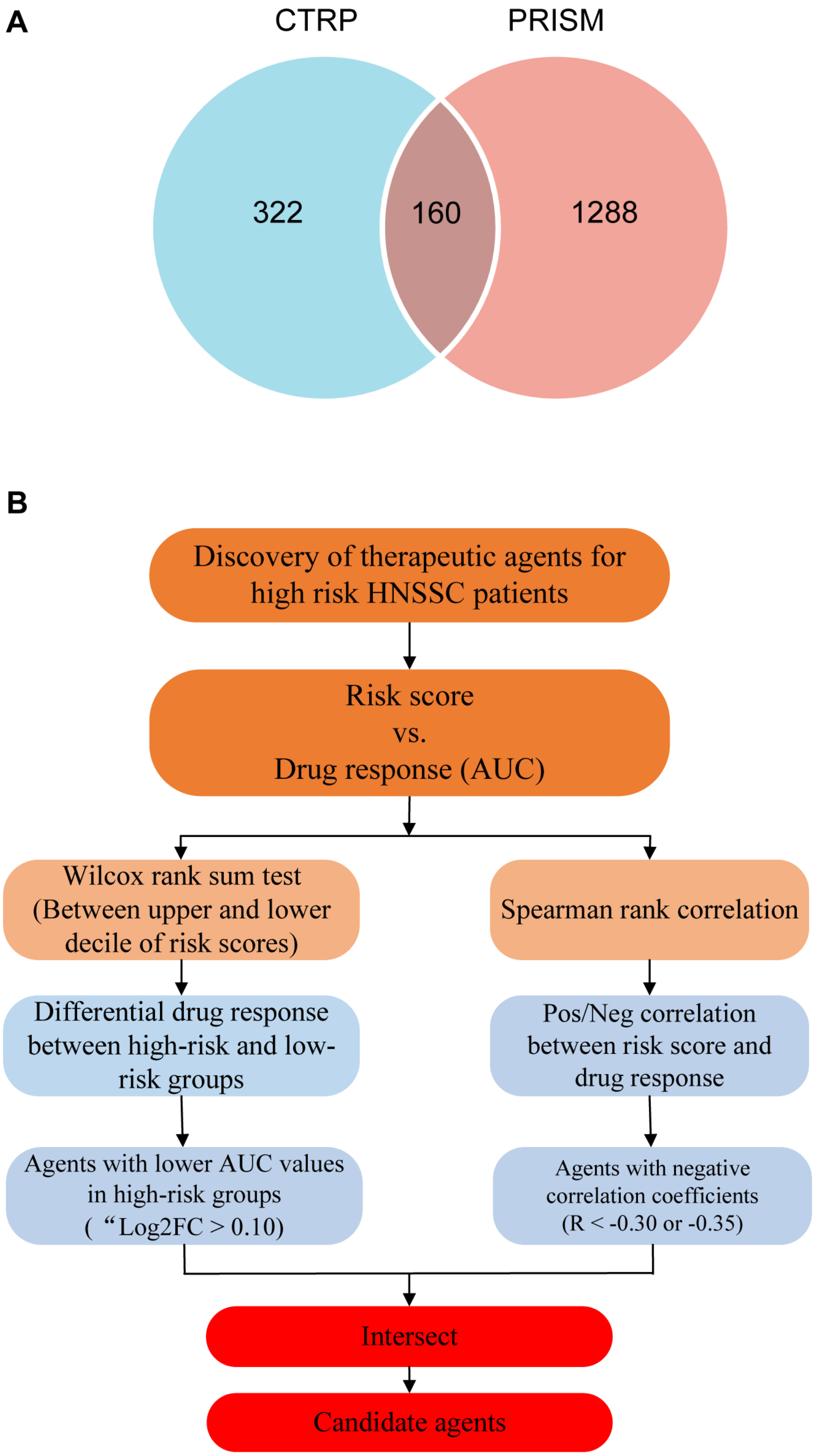

Figure S12. Identification of candidate agents with higher drug sensitivity in

high-risk score patients with HNSCC. (A) Venn diagram for summarizing included compounds from CTRP and PRISM datasets. (B) Schematic outlining the strategy to identify candidate agents with higher drug sensitivity in high-risk score patients with HNSCC.
